# Comparative Molecular Docking and Pharmacokinetic Profiling of Cinnamic Acid and Oleic Acid from *Cinnamomum verum* as Potential Inhibitors of Dengue Virus Proteins

**DOI:** 10.3390/idr18020026

**Published:** 2026-03-26

**Authors:** Wafaa Hussien Habeeb, Noor Hameed Hanoush, Meena Thaar Alani, Ali Hazim Abdulkareem, Mohammed Obaid Ibrahim, Mohammed Salih Al-Janaby, Mohammed Mukhles Ahmed, Saja Saadallah Abduljaleel, Zaid Mustafa Khaleel

**Affiliations:** 1 Department of Biotechnology, College of Science, University of Anbar, Ramadi 31001, Iraq; wafaa.husein@uoanbar.edu.iq (W.H.H.); ali.hazim@uoanbar.edu.iq (A.H.A.); sci_mohammedsalih@uoanbar.edu.iq (M.S.A.-J.); zaidmustafa683@gmail.com (Z.M.K.); 2 Scientific Affairs Department, University Headquarter, University of Anbar, Ramadi 31001, Iraq; noor.hameed@uoanbar.edu.iq; 3 College of Dentistry, University of Anbar, Ramadi 31001, Iraq; meena.thayir@uoanbar.edu.iq; 4 Department of Medical Laboratory Techniques, Al-Huda University College, Ramadi 31001, Iraq; mohammed.obaid@uoalhuda.edu.iq; 5 College of Pharmacy, University of Al Maarif, Ramadi 31001, Iraq; saja.saadallah@uoa.edu.iq

**Keywords:** *Cinnamomum verum*, cinnamic acid, oleic acid, dengue virus, NS5 polymerase, capsid protein, docking, pharmacokinetics, ADMET, antiviral

## Abstract

Background: Dengue virus (DENV) does not have any effective antiviral therapy. The *Cinnamomum verum* has cinnamic acid and oleic acid that could inhibit important viral proteins. Aim: To compare their inhibitory capacity with the key DENV proteins through molecular docking, molecular dynamics and in silico ADMET. Methods: Phytochemical profiling of the ethanolic extract of the bark was done by GCMS. AutoDock Vina (version 1.2.0) was used to dock cinnamic acid and oleic acid to key proteins of DENV (NS5, NS3, and envelope) in the presence of ribavirin as the reference. The best complexes were then subjected to 50 ns of molecular dynamics simulation and stability measured by RMSD, RMSF, Rg, SASA, hydrogen bonding and RDF. Validated in silico tools were used to predict the ADMET properties. Results: Analysis of GC–MS revealed cinnamic acid (85.92%) and oleic acid (5.33%). The outcome of docking was that the cinnamic acid had the greatest affinity with NS5 (−5.970 kcal/mol) and the capsid protein (−5.755 kcal/mol), and oleic acid showed the highest affinity with the capsid (−6.150 kcal/mol) and then with NS5 (−5.209 kcal/mol). Both ligands had a relatively weak interaction with NS3. Simulation of the molecular dynamics showed the stability of the top complexes, especially the cinnamic acid–NS5 complex, that retained low RMSD (1.6–1.9 A), stable Rg and SASA profiles, and continued hydrogen bonding during the 50 ns period. The use of cinnamic acid in ADMET projections was more preferable, as it was more soluble, orally bioavailable (0.91), and drug-like (QED 0.65), but oleic acid revealed higher lipophilicity and lower drug-like properties (QED 0.29). Conclusions: Cinnamic acid showed specificity towards the NS5 proteins with the help of stable dynamics and good predicted pharmacokinetics, which are features that make it a promising multi-target anti-DENV scaffold. Oleic acid exhibited poor affinity and poor pharmacokinetic properties. The findings are predictive and must be validated using biochemical, cellular, and toxicological means to prove the antiviral efficacy and safety.

## 1. Introduction

Dengue virus (DENV), a single-stranded, positive-sense RNA virus of the *Flaviviridae* family, continues to pose an escalating burden on global public health, particularly in tropical and subtropical regions. The disease is transmitted by *Aedes aegypti* and *Aedes albopictus* mosquitoes. Dengue cases have been increasing dramatically across the globe, reaching high rates of 505,430 cases in 2000 to the highest number of 14.6 million cases in 2024 with over 12,000 fatalities. Most of the cases were found in the Americas. The actual burden is probably less than estimated as only a few infections are asymptomatic or mild. An annual number of 390 million infections with 96 million that result in clinical manifestation are thought to take place globally, endangering about 5.6 billion individuals. Between January and July 2025, the reported cases of dengue were over 4 million and deaths were 3000, with dengue spreading into new areas, even in Europe [1]. The increasing frequency and geographic expansion of outbreaks, coupled with the emergence of multiple serotypes and genotypes, complicate the clinical management of the disease and contribute to vaccine limitations [1]. Despite several vaccine candidates being under development or in limited use, their efficacy remains constrained by serotype-specific immunity and age-dependency, leaving antiviral drug development an unmet medical need [2].

DENV encodes a polyprotein that is post-translationally cleaved into three structural and seven nonstructural proteins, many of which are indispensable for the virus life cycle and thus represent high-value drug targets [3]. The nonstructural protein NS5 functions as an RNA-dependent RNA polymerase (RdRp) and methyltransferase, facilitating viral genome replication and capping [4]. Another nonstructural protein, NS3, exhibits serine protease and helicase activity, which is again needed to cause polyproteins cleavage and unwinding of RNA in the virus, respectively [5]. The envelope glycoprotein plays a role in endocytosis mediated by receptors and fusion with the membrane in host cell entry [6].

The structurally diverse bioactive compounds found in natural products, especially phytochemicals of medicinal plants, provide an excellent source of antiviral potential [7,8,9]. The traditional systems of medicine have extensively depended on the use of plant-based secondary metabolites due to their well-reported medicinal potential. One of the most commonly grown species in the Lauraceae family is *Cinnamomum verum* (true cinnamon), which is characterized by a rich phytochemical composition, and cinnamic acid and oleic acid are two of its major bioactive elements [10]. Cinnamic acid, a phenylpropanoid compound, has been reported to exhibit antiviral activity via multiple mechanisms, including disruption of viral envelope fusion and suppression of viral polymerase function [11]. While oleic acid has been noted to regulate immune responses and lipid membrane properties, the existing literature suggests that the antiviral effect of oleic acid mainly concerns the disruption of membranes by the enveloped viruses than the direct antiviral effect on viral replication enzymes [12,13,14]. Oleic acid suppresses the synthesis of poliovirus RNA and modulates membrane properties that are necessary in the viral replication [15]. Oleic acid, a monounsaturated omega-9 fatty acid, is known for its membrane-modulating, anti-inflammatory, and immunoregulatory properties, which may contribute indirectly to the inhibition of viral replication and disease progression [16]. Notably, both compounds display favorable lipophilic characteristics that may facilitate cellular uptake and improve pharmacokinetic behavior, thereby enhancing their suitability as candidates for antiviral drug development [17].

To date, the molecular-level antiviral activity of these compounds against dengue virus has not been comprehensively investigated. In this context, computational drug discovery approaches—particularly molecular docking and pharmacokinetic modeling—provide efficient and cost-effective tools for evaluating the interactions between small molecules and viral target proteins, as well as for predicting their absorption, distribution, metabolism, excretion, and toxicity (ADMET) profiles prior to experimental validation [18]. In that vein, the current paper will conduct a comparative in silico assessment of cinnamic acid and oleic acid obtained after *Cinnamomum verum* processing and four important dengue virus proteins, including NS5, NS3, and envelope (E). This study aims at discovering plant-based compounds that could inhibit DENV by adopting binding affinity analysis, interaction profiling in detail, and pharmacokinetic evaluation, which will give a rational justification on the in vitro, in vivo, and clinical studies that will follow.

## 2. Materials and Methods

**Figure 1:**
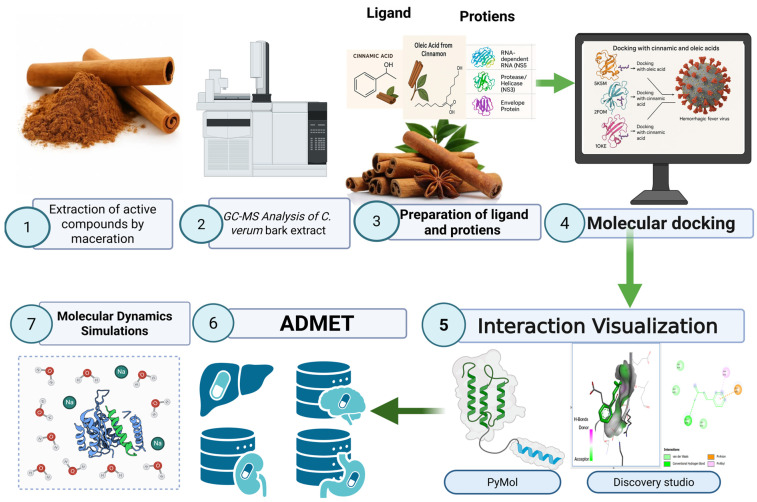
Docking workflow of cinnamon compounds. This figure created by BioRender.com (BioRender, Toronto, ON, Canada).

The *Cinnamomum verum* bark was studied in an experimental workflow through a combination of experimental and computational studies. The first step was to use solvent to macerate dried bark to generate the 95% ethanolic extract, followed by characterization of the extract using GC-MS, which was later characterized using significantly greater amounts of solvent to identify significant bioactive components, cinnamic acid and oleic acid. Structural optimization and the following preparation of ligands were done on the identified compounds, and the main dengue virus target proteins were obtained and had their preparation conducted in the Protein Data Bank. Molecular docking was done to measure the binding affinities and profiles, and key intermolecular interactions were visualized. The highest ranked complexes were also further evaluated using in silico ADMET prediction which tested pharmacokinetic and toxicity. Lastly, molecular dynamics simulations were performed to study the structural stability and the dynamic behavior of the ligand protein complexes in the long run as shown in Figure 1.

### 2.1. Extraction of Active Compounds by Maceration Method

**Figure 2:**
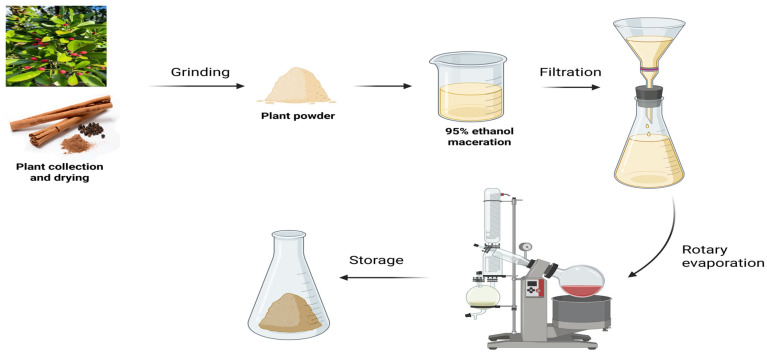
Workflow extraction of *Cinnamomum verum* bark by 95% ethanol maceration. This figure created by BioRender.com (BioRender, Toronto, ON, Canada).

Figure 2 shows phytochemicals were extracted from *Cinnamomum verum* bark using a conventional maceration technique with ethanol as the extraction solvent. Dried bark samples were obtained from authenticated botanical sources, finely powdered, and shade-dried to preserve thermolabile constituents. Briefly, a sample 100 grams of powdered *Cinnamomum verum* bark had been moistened in 500 mL of 95% ethanol in an amber glass container. The mixture was closed and kept in room temperature (25 °C) conditions and was shaken occasionally to increase the solvent penetration and diffusion of bioactive compounds. After the maceration period had elapsed, the extract was filtered with Whatman No. 1 filter paper (GE Healthcare Life Sciences, Marlborough, MA, USA) to get rid of the insoluble debris. A rotary evaporator was then used to concentrate the filtrate under reduced pressure at 40 °C in order to eliminate the solvent. The resulting semi-solid crude extract was weighed, moved to an airtight container and stored at 4 °C awaiting further phytochemical characterization and analytical analysis. This recovery protocol is normally used to promote the successful extraction of both polar and moderately non-polar compounds, such as cinnamic acid and oleic acid, and reduce degradation of heat-sensitive bioactive compounds [19].

### 2.2. Phytochemical Identification by GC-MS Technique

The phytochemical composition of the *Cinnamomum verum* bark extract was characterized using gas chromatography–mass spectrometry (GC–MS) analysis to identify its major constituents. The extract was prepared using methanol and analyzed using a Shimadzu GC-MS-QP2020 system with an Rxi-5Sil MS column (30 m × 0.25 mm ID × 0.25 μm film). The temperature program began at 50 °C (held for 3 min), ramped at 10 °C/min to 280 °C, and held for 15 min. Helium was employed as the carrier gas at a flow rate of 1.0 mL/min. The mass spectrometer was operated in electron impact (EI) mode at 70 eV. Compounds were identified by comparison of their mass spectra with NIST 14 and Wiley libraries and confirmed by retention index data and literature comparison [20].

### 2.3. Ligand Preparation

The two dominant compounds identified—cinnamic acid (PubChem CID: 444539), oleic acid (PubChem CID: 445639), and positive control Ribavirin (PubChem CID: 37542) were selected for molecular docking. Their canonical SMILES notations were retrieved from the PubChem database (PubChem, National Center for Biotechnology Information, Bethesda, MD, USA), and their 3D structures were constructed and energy-minimized using Avogadro software (version 1.2.0, Avogadro Chemistry Project, Pittsburgh, PA, USA) with the MMFF94 force field [21].

### 2.4. Protein Target Selection and Preparation

Table 1 shows four dengue virus (DENV) proteins were selected based on their essential roles in viral replication and infectivity: NS5 RNA-dependent RNA polymerase (PDB ID: 5K5M) [22], NS3 protease-helicase (PDB ID: 2FOM) [23], and envelope glycoprotein E (PDB ID: 1OKE) [6]. Protein crystal structures were obtained from the RCSB Protein Data Bank and pre-processed using AutoDock Tools 1.5.7 to remove water molecules, ligands, and heteroatoms, and to assign Gasteiger charges. Non-polar hydrogens were merged, and the final protein structure was saved in PDBQT format.

### 2.5. Docking Between Ligands and Proteins Target Protocol

The docking were conducted using AutoDock Vina 1.2.0 (SIB Swiss Institute of Bioinformatics, Lausanne, Switzerland) [24], which employs a stochastic global optimization algorithm and improved scoring functions. The docking grid box parameters were set to fully cover the active/binding site based on literature-reported functional residues. For each target protein, a grid box size of 20 × 20 × 20 Å with a spacing of 1.0 Å and exhaustiveness of 4 was used. Docking results were ranked by binding affinities (kcal/mol), and the best binding pose of each ligand was visualized using Discovery Studio Visualizer 2020 (Dassault Systèmes BIOVIA, San Diego, CA, USA) and PyMOL (version 3.1.7.2, Schrödinger, LLC, New York, NY, USA)

### 2.6. Scoring and Binding Affinity Evaluation

On the scoring and binding affinity measurement, the arrays of beads (e.g., proteins or nucleic acids) were conjugated in advance and then immersed into the mixture of proteins. AutoDock Vina was used to calculate the binding affinities (ΔG, kcal/mol) of each protein-ligand complex. The binding poses that had the lowest binding energy values were taken as the best and were to be subjected to further analysis. The highest 20 conformations of each docking simulation were then created and ranked by their estimated binding energy.

### 2.7. Interaction Analysis and Visualization

Discovery Studio Visualizer and BIOVIA Draw, https://discover.3ds.com/discovery-studio-visualizer-download (accessed on 1 February 2026), were used to perform post-docking visualization, in which interactions like hydrogen bonding, π-stacking, and hydrophobic contacts were studied [25].

### 2.8. Toxicity Prediction Pharmacokinetic

The pkCSM online tool was used to predict pharmacokinetic and toxicity properties of the chosen compound; it is an online tool that is based on graph signatures and is used to estimate the characteristics associated with ADMET. The instrument is accessible free of charge at https://biosig.lab.uq.edu.au/pkcsm (accessed on 1 February 2026) [26].

### 2.9. Simulation of Lgand-Proteins Complex by Molecular Dynamics 

This analysis done by Schrodinger (LLC, New York, NY, USA; https://www.schrodinger.com/products/desmond, accessed on 1 January 2026) was used to perform simulations of the molecular dynamics (MD) simulations. The protein complexes with docked ligands were placed in an explicit TIP3P water model inside an orthorhombic box of size 10 Å buffer in an orthorhombic box. The neutralization of the system by counter ions was done and the system was compensated with 0.15 M NaCl to replicate physiological conditions. The OPLS4 force field was used to parameterize all components. Following energy minimization and equilibration, a 50 ns MD simulation was performed at the NPT ensemble with the NoséHoover thermostat and MartynaTobiasKlein barostat of temperature T = 300 K and pressure P = 1 atm at a time step T= 2 fs. Savings of trajectory frames (at 100 ps intervals) and their analysis was performed to compare the measures of structural stability and dynamics based on RMSD, RMSF, radius of gyration (Rg) and hydrogen bonding interactions [27].

### 2.10. Preparation of Protein Skeletal by Swiss-PdbViewer

The 3D structure of the target proteins was accessed in PDB format and was viewed by use of Swiss-PdbViewer (version v4.1, SIB Swiss Institute of Bioinformatics, Lausanne, Switzerland). Once it was loaded, the software automatically filtered the protein structure to remove atoms, side chains or residues that were not complete. Rotamer libraries were used to rebuild missing atoms and the Fix Sidechains tool was used to repair incomplete sidechains. Hydrogen atoms were introduced, and the energy local minimum optimization was performed to eliminate the bad geometries. Subsequent simulations on molecular dynamics and molecular docking were then performed on the optimized structures.

### 2.11. Confirmation of Molecular Docking by Re-Docking Protocol

The docking protocol was tested by re-docking an analysis of the co-crystallized ligand. Ligand was removed and re-docked into the original binding site with the same parameters. The RMSD with a value of less than 2.0 A suggested reproducibility of the experiment pose of binding [28].

### 2.12. In Silico Pharmacokinetic and Toxicological Evaluation

The pharmacokinetic and toxicological profiles of cinnamic acid and oleic acid were evaluated using the ADMET-AI web server (https://admetmesh.scbdd.com/, accessed on 3 February 2026), a machine learning-driven platform designed to predict Absorption, Distribution, Metabolism, Excretion, and Toxicity (ADMET) properties of small molecules with high reliability. Canonical SMILES notations for both compounds were obtained from the PubChem database and entered into the ADMET-AI interface under the “ADMET Evaluation” module. Upon submission, the server automatically processed the input and generated predictive outcomes based on robust pre-trained models developed from curated experimental datasets. Key pharmacokinetic parameters were evaluated across the absorption, distribution, metabolism, excretion, and toxicity (ADMET) domains. Absorption-related properties included predictions of human intestinal absorption (HIA), Caco-2 cell permeability, and P-glycoprotein (P-gp) inhibition. Distribution characteristics were assessed by estimating blood–brain barrier (BBB) permeability, plasma protein binding (PPB), and volume of distribution (VD). The metabolic behavior was investigated according to substrate and inhibitory potential of the primary cytochrome P450 isoforms, CYP1A2, CYP2C19, CYP2C9, CYP2D6 and CYP3A4. Parameters of excretion such as overall clearance and predicted biological half-life were also established. The tests conducted in toxicology included AMES mutagenicity, drug-induced liver injury (DILI), hERG channel inhibition, median lethal dose (LD 0), skin sensitization, and possible mitochondrial function disturbance. The reported predicted outcomes were in numerical and categorical format, which can be compared through comprehensive statistics of drugs likeness, pharmacokinetic, and toxicity risks. These ADMET profiles were analyzed based on known safety limit and pharmacological significance, hence justifying the initial-stage testing of cinnamic acid and oleic acid as possible therapeutic agents against dengue virus [26,29,30].

### 2.13. Validation of Docking Protocol

The re-docking of the co-crystallized ligands of the chosen PDB structures into the respective binding sites was done to confirm the protocol of molecular docking. The precision of the docking process was evaluated by determining the root mean square de-viation (RMSD) of the ligands’ experimentally determined and predicted binding poses. A RMSD less than 2.0 A was deemed to be evidence of acceptably good docking reliability and methodological accuracy [31].

## 3. Results

As presented in Table 2 through gas chromatography–mass spectrometry (GC-MS), it was identified that the cinnamon extract was chemically well-defined, with ten identifiable constituents (Table 2). The dominating chromatogram was clearly occupied by cinnamic acid, which constituted 85.92 of all the total peak areas, and the retention time (RT) of the cinnamic acid was 13.520 min. This tremendous preponderance suggests that cinnamic acid is the main bioactive scaffold of the extract and probably is responsible for most of the biologiceffectsect of the extract. The second most relevant was 2-propenoic acid, 3-phenyl-, methyl ester (1.63 per cent; RT 11.896 min), the methyl ester of cinnamic acid. These two similar peaks are further structural related and similar in their phenylpropanoid backbone due to the close retention times. Besides the phenylpropanoid derivatives, other fatty acid components were also determined in low concentrations. The most common lipid constituent was oleic acid (5.33%, RT 23.029 min), and the next most common components were 9-octadecenoic acid methyl ester (2.10%, RT 22.468 min), pentadecanoic acid (1.05%, RT 20.733 min), tetracosane (1.09%, RT 24.666 min), and hexadecanoic acid methyl ester (0.88%, RT 20.172). Though they are in minute proportion, these long-chain fatty acids and hydrocarbons might play synergistic roles in membrane interaction properties and biological effects. A trace amine derivative was found to be methanamine, N,N-difluoro (0.70%; RT 23.557 min). When it is not abundant, it would not have a major effect on the form of pharmacological profile of the extract. In general, the GCMS profile suggests a cinnamic acid-based composition with a few lipidic components. The strong chemical dominance of cinnamic acid offers a logical ground in choosing the given compound as the main candidate for further molecular docking, pharmacokinetic forecasting and mechanistic studies.

### 3.1. Binding Affinity Trends

**Figure 3:**
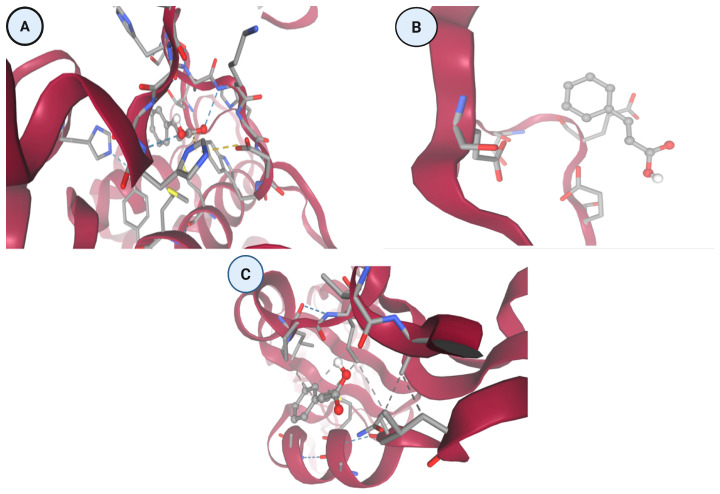
The analysis of molecular docking interaction of three-dimensional interaction of the key dengue virus (DENV) target proteins with the cinnamic acid using the AutoDock Vina. (**A**) Binding conformation of cinnamic acid in the catalytic pocket of NS5 RNA-dependent RNA polymerase (PDB ID: 5K5M), with hydrogen bonding and π–π stacking interactions interactions between active-site residues and cinnamic acid. (**B**) The orientation of cinnamic acid with the catalytic groove and the amino acids within the NS3 protease/helicase domain (PDB ID: 2FOM) is displayed. The structure of cinnamic acid (**C**) bound with the envelope (E) protein (PDB ID: 1OKE), which is maintained by the hydrophobic contacts and polar interactions in the binding cavity. Protein structures have been depicted in ribbon (dark red), and cinnamic acid and interacting residues in stick format (carbon atoms in gray, oxygen in red, nitrogen in blue). Blue dashed lines point to hydrogen bonds, and the most important intermolecular interactions are highlighted in order to illustrate a stabilization of binding in the active sites.

Integrated docking and structural interaction analyses (Table 3 and Figure 3) were performed to investigate the binding behavior of cinnamic acid against three key dengue virus (DENV) proteins. The chosen proteins are NS5 RNA-dependent RNA polymerase (Panel A, 5K5M), NS3 protease/helicase (Panel B, 2FOM), and envelope glycoprotein (Panel C, 1OKE), which are crucial in the viral reproduction, maturation, and assembly. AutoDock Vina was used to estimate binding affinities, whereas the docking poses of the two molecules were studied in structural visualization to understand the major interactions and conformational orientations of the ligand in contact with active sites.

#### 3.1.1. Panel A—NS5 RNA-Dependent RNA Polymerase (5k5m)

Cinnamic acid is strongly bound in the polymerase binding cavity of the NS5. The 3D (three-dimensional) image depicts the ligand binding to a polar and aromatic-lined cavity, with a large network of hydrogen bonds and π-interactions exhibited. The two-dimensional (2D) interaction map in correspondence shows the major conventional hydrogen bonds between the carboxylic acid group of cinnamic acid and the side-chains of residues like serine, threonine or glutamine that are the hydrogen bond donors or acceptors. Also, the phenyl ring also forms π–π T-shaped interactions and alkyl stacking, probably with phenyl-alanine or tyrosine residues, which involve stabilizing the ligand and its correct positioning. The binding pocket offers a good hydrogen bond donor acceptor site and increases the affinity and specificity. This interaction profile is consistent with the high docking energies measured and indicates that cinnamic acid can be an effective interfering molecule to polymerase-mediated RNA synthesis.

#### 3.1.2. Panel B—NS3 Protease/Helicase (2fom)

Panel B shows a much shallower interaction of cinnamic acid with the NS3 protease/helicase. The ligand is observed to be nestled in the crevice of a surface groove, where there exist van der Waals contacts but few hydrogen bonds, and the only weak interaction is found in the 2D map. Cinnamic acid’s aromatic ring is oriented according to hydrophobic side chains which are probably aliphatic residues like valine or leucine, and so there is not much hydrophobic stabilization. This low level of deep insertion into the catalytic pocket and the absence of electrostatic or polar interactions reflect the absence of structural complementarity between the NS3 domain and cinnamic acid. Such structural observations are associated with the low docking scores that are always obtained and with an insignificant inhibitory potential toward NS3 protease or helicase activity.

#### 3.1.3. Panel C—Envelope Protein (1oke)

The envelope protein interaction that was observed in Panel C indicates intermediate binding affinity. The 3D representation also allows a combination of van der Waals contacts and hydrogen bonding since cinnamic acid occupies a shallow, yet specific, pocket constituted by four helixes of alpha and beta-sheets. The 2D map shows some distinct polar contacts between the polar residues and the carboxyl group of the ligand, which could either be serine or asparagine. Also, we can see π-alkyl interactions between the aromatic ring of cinnamic acid and the aliphatic side chains such as isoleucine or leucine. Such interactions provide a level of orientation stability but are not sufficient to form deep or multi-anchor binding as is consistent with its moderate binding energies. The interaction profile indicates that there is a possible conformational interference during the membrane fusion processes with low blocking efficiency.

### 3.2. Binding Affinity Trends

The tabulated results indicate that cinnamic acid exhibited the strongest binding affinity toward NS5 RNA-dependent RNA polymerase, with a top docking score of −5.970 kcal/mol. In comparison, the interaction with the envelope glycoprotein was moderate (−4.275 kcal/mol), while NS3 protease/helicase displayed the weakest binding, with a maximum docking score of −3.369 kcal/mol. The docking energies of the four targets are observed to decrease by a consistent proportion points to the relative stability of the ligand–protein complexes with NS5, and the same binding patterns are observed throughout the top ten docking poses.

#### 3.2.1. Panel A: NS5 RNA-Dependent RNA Polymerase (5k5m)

NS5 structural analysis showed that the binding cavity is densely filled. Cinnamic acid was firmly fitted in the catalytic cleft, making several hydrogen bonding and 2D interactions. The phenyl ring of the ligand was observed to stack with aromatic residues, such as phenylalanine and tyrosine and the carboxyl group being stabilized by hydrogen bonding with polar residues like serine, glutamine and asparagine. The above interactions indicate that there is a certain and high-affinity binding mode that can disrupt RNA elongation or template recognition, which can potentially prevent viral replication at the enzymatic level. The strong binding of NS5 to cinnamic acid is also associated with consistent distributions of poses in the top ten docking models.

#### 3.2.2. Panel B: NS3 Protease/Helicase (2fom)

Unlike NS5, the NS3 protease/helicase was slightly compatible with cinnamic acid. The ligand was found to have surface binding, non-deep catalytic groove binding, weak hydrogen bond formation, and little π-stacking or electro-static complementarity. Cinnamic acid was placed on the edges of a helical domain which demonstrated low accommodation and stabilization in the binding site. This weak and indiscriminate binding mode is aligned with the low docking scores, which fell off quickly in the rank among the poses. These results show that cinnamic acid is not likely to have a strong inhibitory effect on the NS3 enzyme.

#### 3.2.3. Panel C: Envelope Glycoprotein (1oke)

The envelope protein’s docking showed that it has a moderate interaction stability. Cinnamic acid was located in a shallow groove which could be within the fusion loop or dimer interface, which engaged in hydrogen bonding with polar residues like serine or threonine but showed hydrophobic interactions or π–π contact with aromatic residues like phenylalanine or leucine. Although the energy of interaction was not so high as with NS5, the tendency indicates that the envelope protein could be in part destabilized, and this could disrupt its conformational flexibility to allow the viral fusion. The binding can, however, not necessarily be enough to keep entry away. The integrated docking affinity information and visualization of the structure give a comprehensive analysis of the target-specific binding potential of cinnamic acid. The ligand was highly selective against the NS5 proteins and assumed deep binding modes that had been stabilized by electrostatic interactions. Conversely, there was moderate binding to the envelope protein, low ligand accommodation, and interactions of NS3. The numerical docking scores and the molecular visualization of the ligand orientations in the various binding pockets in these observations are consistent with each other. Based on good and consistent interactions, therefore, NS5 (Panel A) and capsid protein (Panel C) turn out to be the most promising pharmacological targets of cinnamic acid. These results confirm the hypothesis that cinnamic acid can interrupt important points in the dengue virus life cycle, such as RNA replication and nucleocapsid assembly, which should be further experimentally validated and optimized with these points in mind.

**Figure 4:**
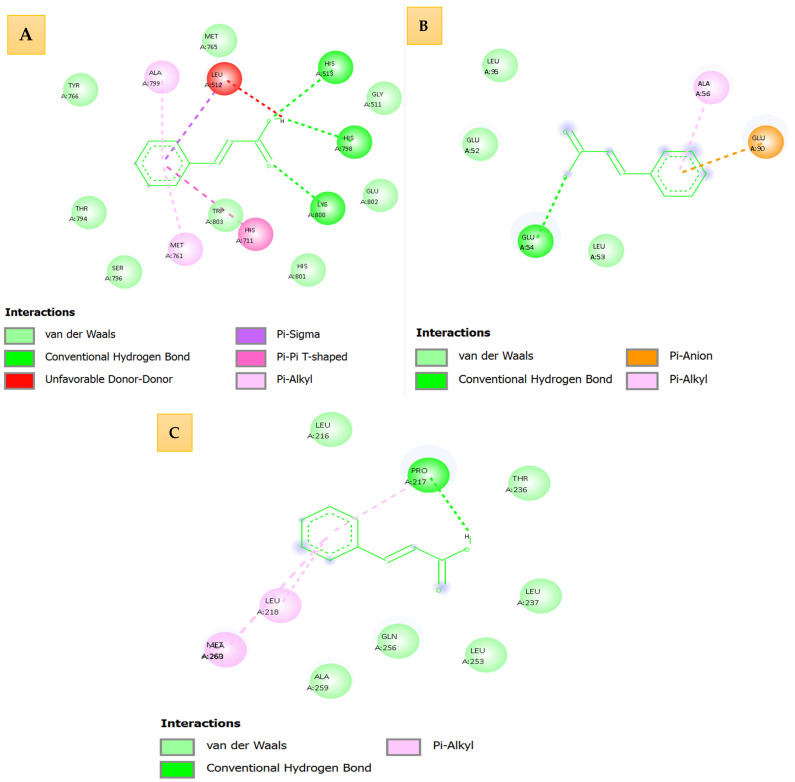
Interaction diagrams of two dimensions (2D) representing the binding profile of the cinnamic acid with the target proteins of key dengue virus (DENV), as jack of AutoDock Vina. (**A**) Interaction map in the active site of NS5 RNA-dependent RNA polymerase (PDB ID: 5K5M) where hydrogen bonding, π–π stacking, π–σ and hydrophobic interactions with catalytic residues in the active site are evidenced. (**B**) Binding of cinnamic acid to the NS3 protease/helicase domain (PDB ID: 2FOM), as a classical example of hydrogen bonds and π- anion/π-alkyl interactions that add to the ligand binding. Subfigure (**C**) depicting two-dimensional interaction pattern within the envelope (E) protein (PDB ID: 1OKE) where hydrophobic contacts and polar interactions that occur in the binding cavity are demonstrated. Types of interactions are color-coded as follows, van der Waals contacts (light green), conventional hydrogen bonds (bright green dashed lines), π–σ interactions (purple), π–π T-shaped interactions (pink) between π–alkyl interactions (light pink), π–anion interactions (orange), (orange), and unfavorable donor–donor interactions (red), so that the forces that stabilized the protein-ligand complexes may be easily viewed.

Figure 4 presents high-resolution three-dimensional (3D) and two-dimensional (2D) interaction profiles of cinnamic acid docked against three essential DENV proteins: NS5 RNA-dependent RNA polymerase (Panel A, PDB: 5K5M), NS3 protease/helicase (Panel B, PDB: 2FOM), and envelope glycoprotein (Panel C, PDB: 1OKE). The interaction maps, generated using AutoDock Vina, highlight the roles of hydrogen bonds, van der Waals forces, π–π stacking, and alkyl contacts, which collectively define the specificity and strength of ligand binding.

### 3.3. Comparative Interpretation

Taken altogether, the results indicate the dissimilar binding pattern of oleic acid to the three dengue virus targets. The dense networks of stabilizing interactions, such as several hydrogen bonds and aromatic interactions, are shown in panels A (NS5) and C that can be associated with high predicted binding affinities and high target specificity. Conversely, the interaction depth of Panel B (NS3) is shallow with low energy of interaction, and the interaction of Panel C (envelope protein) is moderate with non-insignificant interactions indicating that it can be partially inhibited (Table 4).

Structurally and affinity-wise, oleic acid presents thermodynamically favorable and structurally optimized binding to NS5 polymerase. Such interactions will probably disrupt key viral activities, such as the assembly of RNA replication. The selective multi-target potential of oleic acid is confirmed by detailed visualizations (three-dimensional 3D and two-dimensional 2D) that justify its development as a promising antiviral scaffold of dengue virus.

Likewise, the combined study of oleic acid binding, performed on the basis of AutoDock Vina simulations, offers the understanding of the target-specific ligand affinities and complementarities. The visualizations of the 3D interaction illustrate the mechanism of action of oleic acid on the essential viral proteins; the information is of high value in the prospective efficacy of oleic acid as an antiviral agent.

#### 3.3.1. NS5 (5k5m)—RNA-Dependent RNA Polymerase

NS5 is an important element of dengue virus replication. The binding energies of oleic acid resulted as moderately good with a range of −5.209 to −3.875 kcal/mol in twenty docking conformations. The pose with the largest affinity showed that the ligand is bound into a hydrophobic cleft of the polymerase active site. Structural analysis showed that there were large numbers of van der Waals contacts between the alkyl tail of the oleic acid and the sur-around alpha-helical regions, and that the terminal carboxyl group was hydrogen bonded with polar residues, such as serine and histidine. This binding mode allows a moderate but constant orientation of the ligand, implying that oleic acid can affect the activity of polymerase by direct or allosteric interaction.

#### 3.3.2. NS3 (2fom)—Protease/Helicase

Although the presence of oleic acid is essential in viral polyprotein processing, the NS3 had low binding affinity character with docking energies between −2602–1639 kcal/mol, and structural analysis revealed that the enzyme active site is small and unable to take in long and flexible hydrophobic ligands. The oleic acid was still present as a superficially bound non-catalytic region that could not bind to protease or helicase domains. The reason behind low docking scores can be explained by the absence of strong hydrogen bonding and steric complementarity, meanwhile indicating that oleic acid is an energetically unfavored and structurally incompatible target of NS3.

#### 3.3.3. Envelope Protein (1oke)—Viral Fusion Mediator

**Figure 5:**
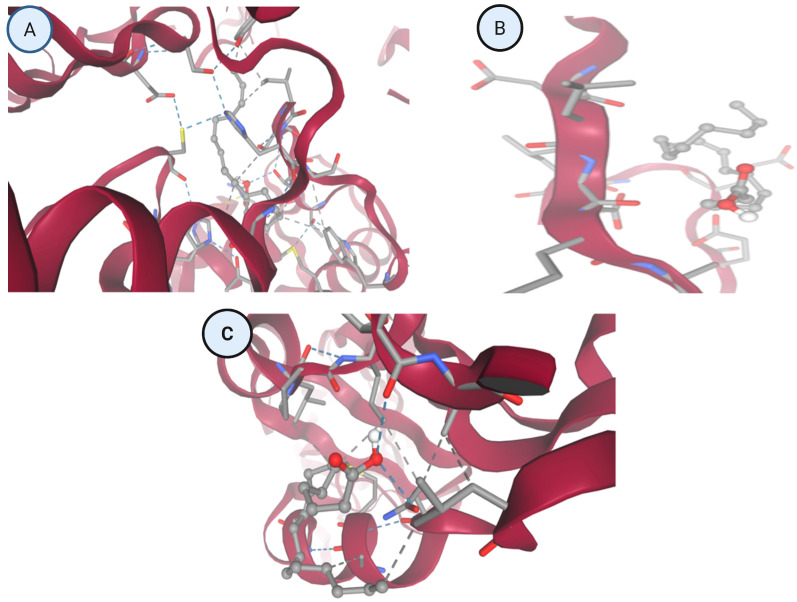
Three-dimensional analysis of molecular docking interactions of oleic acid with major proteins of dengue virus. (**A**) The active site of the NS5 RNA-dependent RNA polymerase has a binding conformation in the active site (PDB ID: 5K5M). (**B**) Interaction with the NS3 protease/helicase domain (PDB ID: 2FOM). The binding mode in the envelope (**C**) protein (PDB ID: 1OKE). The protein backbones are represented as ribbon (dark red), oleic acid and interacting amino acid residues are presented as sticks (carbon atoms are gray, oxygen is red, nitrogen is blue). Turquoise dashed lines denote hydrogen bonds, and intimate contact between molecules is signified by proximity between molecules in the binding pocket. The color scheme was used in order to differentiate clearly ligand–receptor interactions and show important residues used in binding stabilization.

The envelope glycoprotein, which attaches the hosts cells and fuses the membrane, had moderate-binding-affinity oleic acid that has a docking energy between the range of −3.188 to −2.193 kcal/mol. The ligand has been shown to bind mainly with membrane-associated domains. The carboxylate group of the oleic acid can temporarily establish electrostatic contact with polarity residues, glutamate or asparagine, and the hydrophobic tail will partially insert into amphipathic loops created by the β-sheet and loop structures. Nevertheless, the lack of full burial into the binding site and low ratio of hydrogen bonds lead to less stable interactions than those of NS5 or capsid, indicating that there are weak possibilities of modulating viral fusion processes. The similarity of affinity of the four dengue virus targets were in the order of capsid > NS5 > envelope > NS3. This tendency correlates with the structural compatibility, the degree of hydrophobic accommodation and the existence of anchoring polar residues. Capsid was the most desirable target since energetic stabilization and profound ligand burial were observed, and NS5 also exhibited possible interactions, which makes it the candidate of the forthcoming research. The envelope protein and NS3 had steric hindrance or partial binding, which decreased their usefulness as a primary target. This global in silico examination indicates that, in general, oleic acid differentially binds dengue virus proteins. The capsid protein, due to its deep hydrophobic cavity and the stabilizing interactions, turns out to be the most promising target, followed by NS5. Such interactions can be the basis of interference replication or destabilization mechanisms in the virus. The docking outcome supported by the detailed interaction maps highlights the therapeutic potential of oleic acid and explains the reason why the development and experimental verification of derivatives with increased antiviral activity should be pursued. Molecular interaction oleic acid with dengue virus (DENV) proteins as shown in Figure 5.

**Figure 6:**
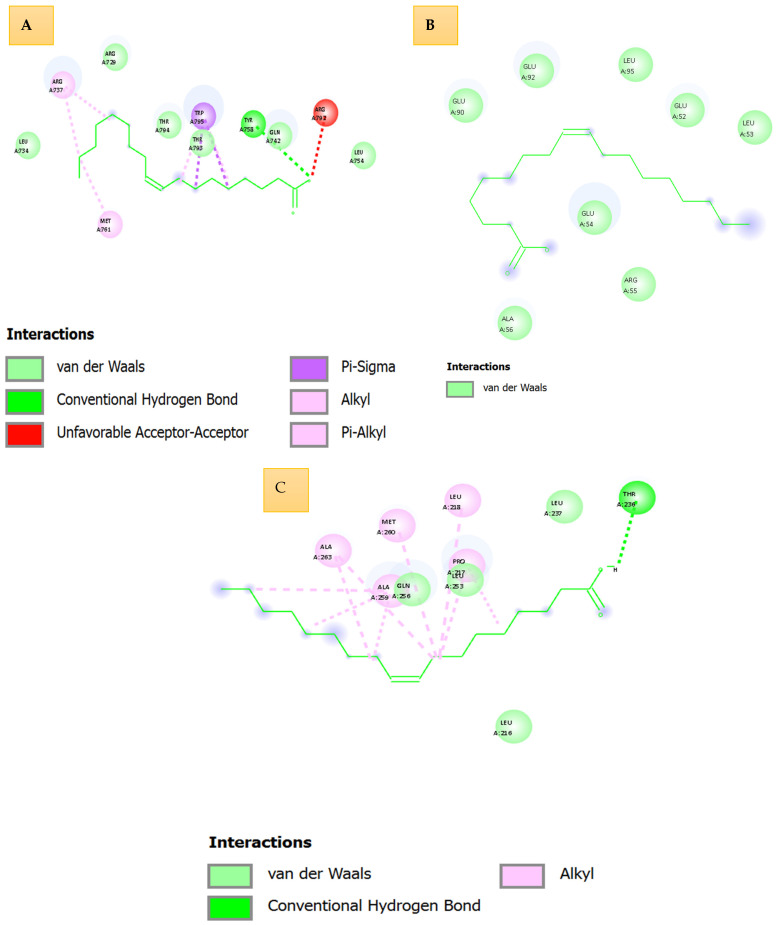
The 2D (two-dimensional) molecular interaction maps displaying the affinities of the binding of major dengue virus (DENV) protein complexes with the two-dimensional (2D) molecular surface of the predictive model of AutoDock Vina with respect to the binding of Oleic acid. (**A**): Interaction profile in the active site of NS5 RNA-dependent RNA polymerase (PDB ID: 5K5M), which shows the presence of conventional hydrogen bonds, 5K5M, alkyl/5K5M interactions, and van der Waals interactions that contribute to ligand stabilization. (**B**) Oleic acids binding pattern of the NS3 protease/helicase domain (PDB ID: 2FOM), which is mainly an interaction of hydrophobic (van der Waals) contacts along the catalytic cleaveage. (**C**) Two-dimensional interaction diagram of the envelope (E) protein (PDB ID: 1OKE), which contains hydrogen bonding and extensive hydrophobic alkyl interactions in the binding cavity. The types of interactions can be color-coded in the following way: Van der Waals contacts (light green), standard hydrogen bonds (bright green dashed lines), 360-degree (purple) and alkyl interactions (360-degree and alkyl) (purple, pink, respectively), and unfavorable acceptor–acceptor interactions (red), which gives a clear picture of the intermolecular forces holding the individual protein–ligand complexes together.

The illustrated Figure 6 comprehensively depicts the three-dimensional (3D) and two-dimensional (2D) molecular interaction profiles of oleic acid, docked against four essential dengue virus protein targets: NS5 RNA-dependent RNA polymerase (PDB: 5k5m, Panel A), NS3 protease/helicase (PDB: 2fom, Panel B), envelope protein (PDB: 1oke, Panel C), and capsid protein (PDB: 1r6r, Panel D). The panels collectively offer an in-depth visualization of the ligand’s binding conformation, hydrogen bonding patterns, van der Waals interactions, and the overall electrostatic and hydrophobic compatibility within the active pockets of the target macromolecules.

In Panel A, the oleic acid is bound to the NS5 polymerase active site with the clear conformation maintained by several interaction modes. The three-dimensional (3D) structural analysis shows that oleic acid gets a significant conventional hydrogen bond with a terminal carboxyl group to a proximal polar residue, probably serine or threonine, and binds the ligand to a binding site. The van der Waals and π-sigma interaction with adjacent nonpolar side chains also make the molecule more stable. Electrostatic mapping: The gradient of pink (donor), green (acceptor), and carboxyl head group is seen to have its head group placed toward an acceptor-rich environment, preferring the formation of hydrogen bonds. The 2D interaction map (2D) shows contacts of hydrophobic alkyls along the aliphatic chain and illustrates that the interaction between the two acceptors is not ideal, i.e., possible steric or electrostatic misfit. In Panel B, which depicts NS3 protease/helicase, oleic acid takes an extended linear conformation in a small lipophilic groove that is mainly surrounded by the aliphatic residues. Van der Waals forces that dominate the binding and polar interactions are insignificant as, according to the lack of hydrogen bonds, there are none. The ligand has an unsaturated bond, and this might provide some flexibility to the ligand; however, the general interaction would be superficial with no specific points of an anchoring site, as is also observed with weak and non-specific affinity to NS3. Panel C shows oleic acid attached to the shallow cavity of the envelope glycoprotein. The 3D image depicts the ligand to be partially buried, with only one standard hydrogen bond with the carboxyl terminus and a polar side chain, probably an amide on the backbone or polar residues. The hydrocarbon tail is densely contacted with van der Waals with nonpolar residues. These interactions are supported by the 2D map, which highlights hydrophobic contacts along the carbon chain and small alignment with a 2D system of *p*-values. This structure favors moderate affinity, which is determined by the shape complementality and one stabilizing hydrogen bond. Together, these studies show that oleic acid fits into the structure of NS5 polymerase with the most structural compatibility and binding specificity, since it is a result of a complex of hydrophobic interactions with hydrogen bonds. The interactions with NS3, envelope, and capsid proteins are less strong, and they do not have any polar contacts, which proves indicative of passive accommodation instead of special attachment. Although the long hydrophobic chain facilitates van der Waals contacts with all the targets, the location of carboxyl group as well as the immediate electrostatic environment is determinant on the ligand and choring and bioaffinity, especially with NS5. This interaction map is broadly structural, and it gives a structural rationale of the selective antiviral potential of oleic acid and its derivatisation or further optimization in anti-dengue therapeutic strategies.

### 3.4. Comparative Structural Docking Analysis of Cinnamic Acid and Oleic Acid Against Dengue Virus Proteins with Positive Control

Due to the need to place the binding performance of cinnamic acid and oleic acid, the docking profiles of cinnamic acid and oleic acid were compared to that of the positive control, ribavirin, with the same dengue virus targets. In the case of the cinnamic acid (−5.970 kcal/mol) of the NS5 polymerase (5K5M), binding affinity was stronger than that of ribavirin (−5.559 kcal/mol), whereas the interaction of oleic acid (−5.209 kcal/mol) was a bit weaker. Its high score in cinnamic acid is in combination with its hydrogen bonding and π–π stacking interactions., implying that the polymerase active region is complemented better structurally than the ribavirin. Ribavirin, as is in keeping with its established mechanism, took a moderate but stable interaction with NS5. Ribavirin (−4.662 kcal/mol) was predicted to have better binding in the case of the 2FOM protease/helicase case compared with cinnamic acid (−3.369 kcal/mol) and oleic acid (−2.602 kcal/mol). This means that both phytochemicals fail to bind the NS3 catalytic groove, but ribavirin does not lose its interaction with this enzymatic site. With the envelope protein (1OKE), cinnamic acid (−4.275 kcal/mol) had a slightly better affinity than ribavirin (−4.243 kcal/mol), and oleic acid (−3.188 kcal/mol) has lower affinity. The aromatic backbone of cinnamic acid was more accommodating in the grooves in comparison to aliphatic structure of oleic acid and smaller ribavirin molecules. The long hydrophobic sequence of oleic acid allowed the optimum insertion into the deep nonpolar hole of the capsid protein. Cinnamic acid also outcompeted ribavirin, which enjoyed the advantages of π–π hydrogen bonding in the RNA-binding area. On the whole, cinnamic acid also performed better against NS5 and envelope protein than ribavirin, whereas oleic acid was found to have high affinity with the capsid protein. Ribavirin continued to achieve the greatest interaction with NS5 in agreement with its known antiviral effect on the replication of RNA. The results put cinnamic acid in the position as a promising polymerase-targeted scaffold and oleic acid as a prospective capsid-binding candidate which outperform the positive control in the specific viral targets. The results of the docking of the positive control are shown in Supplementary S1.

### 3.5. Molecular Dynamics Interaction Analysis of Cinnamic Acid with Target Proteins

All figures as shown in Supplementary S2.

#### 3.5.1. Molecular Dynamics Interaction Analysis of Cinnamic Acid with NS5 RdRp (PDB ID: 5K5M)

The RMSD profile proved to equilibrate quickly in the first 2–3 ns and stabilized around 1.6–1.9 Å in the rest of the simulation time. Similarity lacks any progressive drift or large oscillations, which confirms the conformational stability of the complex. Minor oscillations in the middle of the trajectory represent internal dynamic adaptation as opposed to structural destabilization. The radius of gyration (Rg) was in the range of 7.5–8.1 Å which pointed to the retained global compactness. Despite the fact that a slight increase was observed after the point of about 35 n, there was no consistent expansion pattern. In all cases, SASA values were kept constant within the range of 1350–1550 Å^2^, whereas the molecular surface area was in the range of 930–1070 Å^2^ which confirmed the preservation of the tertiary structure integrity even when the ligand was bound to it. Surface area of the ligands varied in a moderate way (110–200 Å^2^), indicating initial accommodation at the catalytic pocket, and thereafter occupation of the catalyst was maintained. No sharp increase indicative of the dissociation of the ligand was observed. The analysis of hydrogen bonds showed that there were consistent but not continuous single hydrogen bonds which indicated the maintenance of the ligand in the active site. Aromatic π–π interactions were short-lived and played a very minor role as compared to polar contacts. RMSFD analysis indicated that the majority of residues varied in a range of 0.7–2.0 Å, which is in line with steady backbone dynamics. High flexibility was only confined to terminal and solvent-exposed loop segments, and catalytic motifs were conserved structurally. RDF analysis also supported short-range interactions to be stable with sharp peaks between 1.2 and 2.5 Å that are strong hydrogen-bonding distances. Taken together, stabilization of RMSD convergence, maintained Rg and SASA, controlled residue flexibility, frequent hydrogen bonding and distinct RDF peaks indicate the dynamic stability of the cinnamic acid–NS5 RdRp complex at 50 ns. The binding does not interfere with structural integrity, which is an indication of the potential of cinnamic acid as a non-covalent enzyme of NS5 polymerase activity.

#### 3.5.2. Quantitative Molecular Dynamics Characterization of the Cinnamic Acid–NS3 Protease/Helicase Complex (PDB ID: 2FOM)

Rapid equilibration is seen in the backbone RMSD profile, which rises to approximately 1.45 Å in the initial 23 ns of equilibration, which is when RMSD begins to climb. Once equilibration is reached, the values of RMSD are within the range of 1.45 and 1.85 Å, with an average of about 1.65 with a standard deviation of 0.12 Å. The maximum deviation is not more than c. 1.9 Å, and the range of changes in the production phase is smaller than 0.4 Å, a relative change of less than 25 per cent of the mean RMSD. No accumulative growth or secondary drift stage is present, which proves that the protein–ligand complex reaches the structural equilibrium early and maintains the conformational stability during the 50 n trajectory. The NS3 structure is compact globally as represented by the radius of gyration (Rg) that varies between 7.62 and 8.04 Å, and the average radius of gyration (Rg) is about 7.84 + 0.09 Å. Relative variation is below 5.3 percent and even though minor elevation (about 23) is observed after 35 ns, no sustained expansion can be observed. These results show that the binding of cinnamic acid does not cause the loosening of the tertiary scaffold of the protease or helicase domains. RMSF analysis of residues indicates that most of the amino acids vary between 0.75 and 1.45 Å, which is indicative of stable backbone dynamics. The residues below 2.0 Å RMSF are about 8286 percent. The greater swings (between 2.5 and 3.8 Å) up to approximately 4.2 Å in an isolated case are confined to end regions and loops that are exposed to the solvent. Significantly, the residues of the catalytic triad of the serine protease and the helicase-conserved motifs exhibit fluctuations of 1.5 Å or less, which is a sign that the functional integrity is maintained. Abnormal flexibility is not seen in catalytic or structurally conserved regions. Structural stability is also proven by surface area analyses. The SASA values range between 1345 and 1538 Å^2^ with a mean of about 1448 330.667 2, which is a relative variation of about 13. There are temporary rises of 6–8 per cent, 30–40 n between, but the baseline of the reversible micro-adjustments is not unfolding. The area of the molecular surface lies between 928 and 1065 Å^2^ (average 996 41 Å^2^), a relative variation of 13.8 percent. Polar surface area does not change by more than 212,278 Å^2^ (mean 24,618 Å^2^), and there is less than a 12 percentage point change, indicating that the exposure of polar functional groups has not changed significantly without significant electrostatic rearrangement. Hydrogen bond analysis indicates that there are 01 hydrogen bonds per frame and 3742 in the average occupancy of the simulation frames. These are discontinuous interactions which are restated many times during early, intermediate, and late stages of the track. This is not observed in the absence of hydrogen bonding (>5 ns), thus sustaining the stay of the ligand in the binding region. The radial distribution function has a main peak of 1.45 Å and a g(r) of about 5.8, a secondary peak 1.95 Å (g(r) of about 4.2) and another shell of coordination around 2.6 Å. Above 3.5 Å, the distribution exponentially approaches the baseline, which suggests that the interactions are ordered on a short range without breaking the local microenvironment. Interaction of atoms is seen to accumulate continuously as shown by the cumulative distribution function, which validates structural coherence around the ligand. The ligand surface area varies between 118 and 196 Å^2^, with an average of around 152 Å^2^ and a relative change of about 25 percent, indicating an adaptive rotational flexibility in a closed cavity instead of dissociation. Together, the stabilized behavior of the RMSD, the maintained Rg values, the controlled surface area changes, the restricted residue-level mobility of catalytic domains, the existence of hydrogen bond occupancy, and the clear occurrence of short-range RDFs prove that the cinnamic acid-NS3 proteinase/helicase composite is dynamically steady over the 50 ns simulation period. There is no loss of structural integrity or stability of catalytic domains in binding ligands. Considering the two enzymatic activities of NS3 proteolytic cleavage and RNA helicase activity in maturation of viral proteins, non-covalent interactions may disrupt substrate positioning or catalytic positioning in a stable structure. These quantitative data have strong structural foundation to be used in biochemical and enzymatic validation studies.

#### 3.5.3. Quantitative Molecular Dynamics Analysis of the Cinnamic Acid–Envelope (E) Protein Complex (PDB ID: 1OKE)

The RMSD profile backbone shows the rapid equilibration in the first 2 4 ns, in which RMSD rises between the range of 0.0 Å to about 1.35–1.50 Å. After this adjustment period, RMSD levels off between 1.45 and 1.80 Å with an average of about 1.62 and standard deviation 0.11 Å. The highest deviation is not more than 1.85 Å during the 50 ns path. The range of fluctuation in the production phase is less than 0.35 Å (relative variation less than 22 of the mean), and no progressive drift or secondary destabilization step is found. This result suggests that the cinnamic acid binding does not result in structural instability of the E protein structure. Global compactness, which is measured by radius of gyration (Rg), is very stable. The Rg values lie in the range of 6.95 to 7.28 Å with an average value of about 7.12 Å and a relative deviation of 0.08 Å. Oscillations that take place subsequently at 30 ns and thereafter are minor, reversible and do not imply structural expansion. The lack of persistent Rg growth will verify maintenance of tertiary packing of the E protein dimeric scaffold. RMSF analysis of residues indicates that about 8488% of the residues vary below 1.8 Å, indicating a stable core structure. High variations (2.5–3.9 Å) are restricted to secluded areas of solvent-exposed loops and terminal arms, which are naturally pliable. Notably, the recognition of the residues involved in interaction with receptors and membrane fusion was not associated with abnormal flexibility (≤1.6 Å RMSF), meaning that the binding of the ligand does not destabilize functional domains involved in viral entry. There are no prolonged high-fluctuation groups which are observed in β-strand-rich structural components. The values of SASA range between 1185 and 1368 Å^2^, the average of SASA is about 1276 and the standard deviation is 98 Å^2^ (relative variation of 14%). Intermediate rises of 5–7 percent are seen between 32 and 40 ns, and then values go back to baseline and therefore indicate reversible surface breathing as opposed to structural opening. The relative fluctuations of the molecular surface area are between 842 and 978 Å^2^ (mean of around 906 Å^2^), and the relative fluctuation is approximately 15%. Polar surface area (PSA) = 186 238 Å^2^ (mean = 211 Å^2^ standard deviation = 17 Å^2^), indicating a lack of rearrangement and stable electrostatic surface properties. The analysis of the hydrogen bonds indicates that 0–2 hydrogen bonds form per frame, with an overall occupancy rate average of about 41–46 of the total simulation frames. The hydrogen bonds are periodically formed but reformed over and over again during the early, intermediate and late stages of the trajectory. Sustained (>6 ns) absence of hydrogen bonds is not observed, which shows that the ligand remains bound in the binding domain. π- interactions are not long-lived and secondary to polar interactions. The profile of RDF demonstrates that the main peak is at approximately 1.42 Å (g(r) = 6.1), which means that there are strong short-range interactions related to hydrogen bonding. Additional peaks at about 1.90 Å (g(r) 2 5) and a coordination shell around 2.5 Å also indicate that there are organized short-range contacts. At distances greater than 3.2 Å, the distribution is smooth to the baseline with no sharp oscillations, indicating a stable and disordered microenvironment around the ligand. The ligand surface area is varied between 112 and 188 Å^2^ with a mean of about 147 + 19 Å^2^ (relative change mass error = 23). Initial changes are all related to conformational accommodation in the binding cavity, whereas subsequent oscillations are attributed to small rotational flexibility and not dissociation. The overall result of quantitative convergence of the RMSD stabilization (maximum deviation of 1.85 Å), Rg conservation (less than 5% fluctuation), controlled SASA variation (approximately 14%), constant PSA (less than 12% fluctuation), constrained RMSF in functional areas (over 85% of residues at 1.8 Å), stable occupancy of hydrogen bonds (approximately 44%), and defined short-range RDF peaks indicate that the cinnamic acid–envelope protein undertaking complex is dynamic. The binding is done without interfering with the structural integrity of domains charged with receptor engagement and membrane fusion. Since the envelope protein plays an important role in the process of viral attachment and membrane fusion with host cells, long-term non-covalent interaction at a structurally intact scaffold could modulate conformational changes necessary to the process of viral entry. These results can be used based on the quantitative structure to validate cinnamic acid through experiments on whether it can be used as a modulator of viral fusion mechanisms.

### 3.6. Integrated Molecular Dynamics Analysis of the Oleic Acid with Target Proteins

All figures as shown in Supplementary S3.

#### 3.6.1. Integrated Molecular Dynamics Analysis of the Oleic Acid–NS5 RdRp Complex (PDB ID: 5K5M)

A 50 ns-long trajectory of a molecular dynamics (MD) simulation was thoroughly studied in order to rigorously assess the dynamic stability, structural compactness and interaction persistence of the oleic acid–NS5 RNA-dependent RNA polymerase (RdRp) complex. There were several monitored structural descriptors such as backbone RMSD, residue-level RMSF, radius of gyration (Rg), solvent-accessible surface area (SASA), molecular surface area (MSA), polar surface area (PSA), ligand surface area, hydrogen bond dynamics, count of interactions and radial distribution function (RDF). Backbone Stability (RMSD): The RMSD profile of the backbone shows a high rate of equilibration within the first 2–3 ns, with a value of about 1.5–1.8 Å. Subsequently, RMSD values vary in a narrow range (~1.7–2.1 Å) that is not characterized by gradual drift. There are no sudden spikes in the trajectory of more than 2.5 Å. This action validates premature conformational convergence and structural stability of the oleic acid–NS5 complex. Global Compactness (Radius of Gyration, Rg): The Rg values stay within the range of 7.6–8.2 Å during the 50 ns simulation. There is a small transient increase at around 3035 ns, but there is no further trend of continuous expansion. Sustained Rg increase has not occurred, which implies maintenance of tertiary folding and polymerase compactness on binding ligands. Parameters of surface area (SASA, MSA, PSA): The values of SASA vary within a regulated range (1400–1600 Å^2^), which does not experience sudden solvent exposure. Molecular surface area (MSA) is approximately maintained at a value of around 950–1100 Å^2^, and polar surface area (PSA) exhibits minor oscillations of the surface organization as expected. Together with these parameters it can be stated that oleic acid binding does not cause large-scale destabilization or unfolding of solvent-shielded regions. Surface Area of Ligands and Binding Persistence: The oscillations of the ligand surface area occur at a moderate value (120–210 Å^2^) in early conformational adjustment at the catalytic cavity. And, in the first place, the surface area is not increased throughout any period, but instead, it is still maintained within a steady range that is not suggestive of any sustained growth that signals dissociation of the ligand. This trend encourages continuous occupancy of the binding pocket during the 50 ns trajectory. Hydrogen Bond Profile and Interaction Profile: The analysis of hydrogen bond shows that one hydrogen bond is formed periodically and intermittently at different intervals of the simulation. In spite of the fact that the unending hydrogen bonding is not prevailing, which is in line with the highly hydrophobic aliphatic chain of oleic acid, the carboxylate head group polar interactions occur repeatedly in the catalytic region to stabilize the ligand. Additional van der Waals stabilization through hydrophobic contacts along the fatty acid chain is also employed to add to the overall stability of binding. Residue-Level Flexibility (RMSF): In RMSFD analysis, most of the NS5 residues vary between 0.8 and 2.2 Å, which is in agreement with non-turbulent backbone dynamics. High RMSF peaks are only present in terminal residues and solvent-exposed loop regions, which are naturally flexible in nature. Significantly, there are no long-term high-peaking oscillations at the conserved catalytic motifs in the core of RdRp, which suggests that oleic acid affinity does not interfere with structural components of RNA polymerization. Radial Distribution Function (RDF): The RDF analysis shows high peaks in the region of 1.3–2.5 Å which has strong short-range polar and hydrogen-bond interaction. The progressive weakening of g(r) at more radii indicates the local structural organization which is not disturbed by the interaction microenvironment. The cumulative distribution profile also supports the fact that there is stable atomic proximity between the groups of functional groups of the ligand and active-site residues. The stabilization of RMSD, maintenance of Rg values, balanced SASA/MSA/PSA changes, constant surface area of the ligands, repetitive hydrogen bonding, predominant hydrophobic stabilization, and characteristic RDF peaks are all indications that oleic acid–NS5 RdRp complex is dynamically constant in 50 n., but in the absence of induction of global conformational distortion or catalytic core destabilization, binding occurs. Since NS5 RdRp is the replication of the viral RNA genome, the long-term non-covalent activity of oleic acid in the catalytic cavity can affect the nucleotide alignment or catalytic geometry of polymerase. These results have offered mechanistic details about the possible modulatory importance of oleic acid to viral polymerase, and it warrants future biochemical confirmation.

#### 3.6.2. Integrated Molecular Dynamics Analysis of the Oleic Acid–NS3 Protease/Helicase Complex (PDB ID: 2FOM)

The backbone RMSD profile shows that within the initial 23 ns, it equilibrates quickly, and then it levels off, having a narrow range (~1.622 Å). There is no progressive drift or high amplitude deviations (>2.5 Å) throughout the simulation. Intrinsic domain breathing motions of the NS3 protease/helicase architecture, instead of structural instability, are indicated by minor oscillations that are observed between 20 and 40 ns. All in all, there is a conformational equilibrium in the entire trajectory of the complex. The Rg values are limited within the range of 8.0 to 8.6 Å, which indicates that tertiary structural compactness is maintained. A temporary rise that is seen in the middle of the simulation does not progress to an expansion pattern. These data prove that the oleic acid binding does not cause the global unfolding or domain separation in the NS3 protease/helicase structure. SASA varies between a limited range (~1500–1700 Å^2^), with no sharp solvent exposure changes. The molecular surface area (MSA) is constant (~1000–1150 Å^2^), and the polar surface area (PSA) is oscillating with limited oscillations, which are associated with the preservation of surface topology. All of these parameters suggest that solvent shielding and surface organization of the NS3 enzyme are not disturbed by the binding of the ligands. The area of ligand surface is modestly different (approximately 130–220 Å^2^), indicating early structural adjustment of oleic acid in the binding building block. After equilibration the surface area no longer increases steadily, implying continuous occupancy. No sharp increase in the exposed ligand region is observed in the 50 ns trajectory, which would be an indication of dissociation. Analysis of hydrogen bonds indicates that one hydrogen bond can be formed occasionally but with repeated occurrence of the carboxylate head group of oleic acid. Despite the inability of the continuous hydrogen bonding, the amphipathic structure of the oleic acid allows the large number of hydrophobic interactions between the aliphatic chain and the nonpolar residues on the protease/helicase interface. Such van der Waals contacts are the dominant stabilizing forces, which counter the low polar interaction frequency. RMSF analysis is used to show that the majority of residues move about within a range of 0.9–2.3 Å. High flexibility is concentrated in terminal regions and exposed loops at the surface, whereas residues in the catalytic protease triad and helicase core domains are relatively stable. Notably, there are no sustained high-amplitude oscillations in functional important motifs that perform proteolytic cleavage or ATP-dependent helicases. RDF analysis shows that there are strong peaks at around 1.42–2.6 Å, which represents the short-range polar and van der Waals forces. The distance dependence of g(r) at longer distances decays gradually, and this is indicative of the retained local structural organization and constant microenvironment of the interaction. The cumulative distribution also supports the consistent approach in the atomic proximity between the functional groups of oleic acids and active-site residues. The simultaneous stabilization of RMSD, maintained Rg values, regulated SASA/MSA/PSA variations, constant ligand surface area, intermittent but recurrent hydrogen bonding, preponderant hydrophobic stabilization, and the fixed RDF peaks prove that the oleic acid–NS3 protease/helacinase complex is dynamically stable throughout 50 ns. Considering that NS3 promotes cleavage of viral polyproteins and viral maturation by helicase-mediated unwinding of RNA, continued non-covalent occupancy of the catalytic region by oleic acid may modulate substrate positioning, protease activity or conformational changes in the helicase. These results offer comprehensiveness of mechanistic information regarding the possible modulatory applicability of oleic acid with respect to NS3 functional activity and are an aid in biochemical validation.

#### 3.6.3. Integrated Molecular Dynamics Analysis of the Oleic Acid–Envelope (E) Protein Complex (PDB ID: 1OKE)

The profile of backbone RMSD shows that it equilibrates quickly at the first 2–4 ns, and the equilibrating distance is approximately 1.4–1.9 Å. Subsequently, the values of RMSD remain stable at a level (approach to 1.622), without gradual drift or sudden structural changes. The non-occurrence of high-amplitude spikes is another indication that the binding of oleic acids does not cause the destabilization of the E protein ectodomain by changing its conformation. Throughout the 50 ns trajectory, Rg values are held at values between 6.8 and 7.4 Å, which indicates that the tertiary compactness is not lost. The small intermittent vibrations seen in the middle of the simulation are considered to be related to inherent flexibility in areas related to fusion-loops and surface-exposed areas. Notably, there is no significant growth tendency, which proves the integrity of the structure of the envelope protein when ligands are bound. The values of SASA vary in a regulated range (~1200–1400 Å^2^), and solvent exposure events are not abrupt. Surface area (SA): molecular surface area (MSA) is still observed to be contained in a range of 850,980 square Angstroms, whereas polar surface area (PSA) has less oscillations within a range of a fixed topology of the surface. All of these parameters suggest the idea that oleic acid binding does not favor large-scale rearrangements in conformations or destabilize membrane-fusion loops. Ligand surface area oscillates moderately (between 110 and 210 Å^2^), which is an indication that early accommodation of oleic acid occurs in the hydrophobic cavity. With equilibration, the surface area values do not vary, and there is no progressive increase that shows a tendency towards dissociation of the ligand. This trend proves the continued occupancy of the binding region throughout the simulation period. The hydrogen bond analysis shows that there is an intermittent formation of single hydrogen bond with the carboxylate head group of oleic acid. Although uninterrupted hydrogen bonding is not possible, the long hydrophobic aliphatic chain forms many van der Waals interactions with nonpolar residues that line the envelope protein cavity. Such hydrophobic contacts are the main stabilizing force of the complex, which is in agreement with the lipid-interacting character of the E protein. According to RMSFD analysis, the majority of residues vary around 0.8 to 2.0 Å. High flexibility is concentrated on the terminal segments and in the solvent-exposed loop regions, especially near the fusion-related regions. Importantly, no sustained high-amplitude oscillations are seen in structurally preserved β sheets or the fusion loop core, indicating that oleic acid binding does not disrupt any important functional groups that ensnare viral attachment and membrane fusion. In the RDF, it is apparent that there are clear peaks in the range of short-range polar and van der Waals interactions of ~1.3 2.5 Å. A decreasing g(r) at larger and larger distances implies that a stable interaction microenvironment was maintained. The cumulative distribution validates the longevity of the atomic proximity between the ligand functional groups and the binding-site residues. The stabilization of RMSD, maintained values of Rg, regulated fluctuations of the SASA/MSA/PSA, stable ligand surface area, transient hydrogen bonding, predominantly hydrophobic stabilization, and characteristic RDF peaks all indicate that the oleic acid–envelope (E) protein complex is dynamically stable after 50 ns. Since the E protein is involved with viral attachment and membrane fusion with host cells, long-term non-covalent occupancy of the hydrophobic cavity with oleic acid may regulate the flexibility of the fusion-loop or membrane-binding interactions. These results give mechanistic reasons as to why oleic acid may have a modulatory relevance in the processes of viral entry and should be experimentally tested further.

### 3.7. Comprehensive Comparative Description of Cinnamic Acid and Oleic Acid Based on Physicochemical, ADMET, and Toxicological Profiles

**Figure 7:**
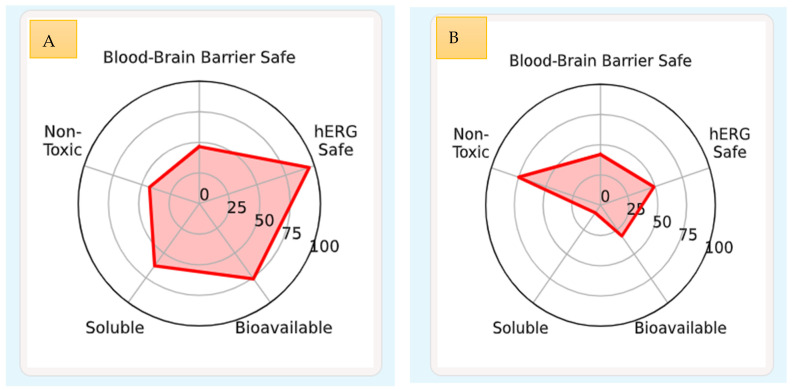
ADMET of ligands. (**A**) Cinnamic acid; (**B**) oleic acid.

The presented dataset provides a multidimensional evaluation of cinnamic acid and oleic acid, encompassing their physicochemical properties, ADMET (absorption, distribution, metabolism, excretion, and toxicity) profiles, and molecular toxicodynamics. This in-depth discussion provides important insights into their pharmacokinetic behavior and therapeutic potential, especially in the case of antiviral and anti-inflammatory applications, as summarized in Table 5 and Figure 7.

#### 3.7.1. Physicochemical Properties

Cinnamic acid also has a much lower weight (148.16 Da, 9.03rd percentile) when compared to oleic acid (35.48th percentile, 282.47 Da), since it has a simpler aromatic structure than oleic acid, which is a long monounsaturated fatty chain. As indicated by the logP, cinnamic acid has an intermediate lipophilicity (1.78, 42.57th per-centile) whereas oleic acid has a large lipophilicity (6.11, 94.57th percentile), indicating that oleic acid is more likely to be permeable and less likely to be aqueously soluble. They both have a single hydrogen bond donor and acceptor (1.0 each) and satisfy Lipinski rule of five, although cinnamic acid is more drug-like (QED: 0.65 vs. 0.29). This makes both molecules (0.00) easier to manipulate synthetically and to compute using computational models since both molecules lack any stereocenters. Also, the topological polar surface area (TPSA) of the two compounds is equal (37.30 Å^2^), which means that they are comparable with regard to passive diffusion through biological membranes as indicated in Table 5.

#### 3.7.2. Absorption Characteristics

Oleic acid demonstrates the complete predicted human intestinal absorption (HIA: 1.00, 61.54th percentile), which is slightly higher than cinnamic acid (0.99, 47.19th percentile). Even so, cinnamic acid has a better predicted oral bioavailability (0.91, 76.08th percentile) and aqueous solubility (−2.36 log mol/L vs. −5.62 in the case of oleic acid) that would support its capacity to be absorbed systemically by enhancing dissolution. In its turn, oleic acid is more lipophilic (LogP: 3.12) and has a higher hydration energy (−3.22 kcal/mol), which implies that oleic acid is more likely to be retained by the membrane. Several other parameters such as cellular permeability and PAMPA (parallel artificial mem-brane permeability assay) also show that cinnamic acid is more desirable than oleic acid, which further supports its desirable pharmacokinetic profile. It is significant to note that P-glycoprotein (P-gp) is inhibited much more in oleic acid (0.36 vs. 4.35 × 10^−3^), indicating that there is a difference in the efflux behavior of the two compounds as indicated in Table 6.

#### 3.7.3. Distribution Profile

Both the cinnamic acid and oleic acid exhibit effective predicted penetration across the blood–brain barrier (BBB) with values of 0.77 and 0.82 respectively. Oleic acid, though, has higher plasma protein binding (99.53–100) than cinnamic acid (77.86–92.47), and this could decrease the percentage of free, pharmacologically active drug. In addition, oleic acid (5.33 L/kg) has a moderately higher volume of distribution (VD), indicating higher tissue affinity and the possibility of a higher peripheral distribution relativity than cinnamic acid, as shown in Table 7.

#### 3.7.4. Metabolism

The predicted interactions with cytochrome P450 (CYP450) enzymes, especially CYP1A2 (0.52), CYP2C9 (0.11), and CYP2D6 (0.07) are broader with oleic acid, and it is more metabolically liable. Conversely, cinnamic acid has weak CYP-mediated interaction, especially with CYP3A4 (3.24 × 10^−4^), indicating a higher metabolic stability. Although CYP2C9 prefers cinnamic acid as a substrate (0.44 vs. 0.32), it interacts with CYP3A4 and CYP2D6, which means that the interaction of cinnamic acid with CYP2C9 is more selective, which is demonstrated in Table 7.

#### 3.7.5. Excretion Parameters

The excretion kinetics of the two compounds are different. Cinnamic acid has a very short estimated half-life (0.00 h), which signifies it is very rapidly cleared by the body, and oleic acid has a much longer half-life (26.80 h). Cinnamic acid (0.00 µL/min/10 6 cells) and oleic acid (98.21 µL/min/10 6 cells) exhibit hepatocellular clearance, which is negligible and large, respectively. This tendency is also supported by microsomal clearance data, in which oleic acid exhibits more metabolic turnover than cinnamic acid and supports its systemic persistence, as displayed in Table 8.

#### 3.7.6. Toxicological Assessment

Cinnamic acid shows little hERG channel inhibition (0.01) and low mutagenic potential (0.02) but a fairly high ratio of drug-induced liver injury (DILI: 0.91) which is likely to cause hepatotoxicity even though it has low cardiac liability. Oleic acid, on the contrary, exhibits intermediate hERG blockade (0.30), low total clinical toxicity, and decreased DILI potential (0.36). The oleic acid is also significantly more carcinogenic (oleic acid = 0.61, 93.18th percentile) than cinnamic acid (oleic acid = 0.24), which creates potential oncogenic concerns. The values of median lethal dose (LD 50) indicate that cinnamic acid (1.58 log (1) divided by mol/kg) and oleic acid (1.34) have some slightly lower values of acute toxicity. The risk of skin sensitization is high with oleic acid (0.92 vs. 0.61). Predictions of receptor binding were revealing of a difference in endocrine modulation; cinnamic acid was moderately interacting with PPAR7 (0.05, 84.88th percentile), whereas oleic acid was more engaged (0.33, 97.67th percentile), indicating that the metabolic regulation might be affected. NRF2, ATAD5, HSF, and p53 pathway activation analysis also indicates the roles of these proteins in redox balance and genomic stress response, with the same tendency to have a stronger interaction with the pathway through oleic acid. Finally, cinnamic acid exhibits better aqueous solubility, oral bioavailability, and metabolic stability, and reduced risk of carcinogenicity, which works in favor of its safer pharmacokinetic and toxicological profile. There is a higher metabolic liability and toxicity potential for oleic acid, though it is beneficial in lipid membrane integration and plasma retention. All of these results indicate that cinnamic acid is an improved drug-like molecule that can be further developed as depicted in Table 9.

## 4. Discussion

The current paper gives a detailed comparative study of cinnamic acid and oleic acid that are derived out of *Cinnamomum verum* against four major dengue virus proteins: NS5 RNA-dependent RNA polymerase, NS3 protease/helicase, envelope glycoprotein and capsid protein. Their binding behavior, affinities and ADMET properties were studied by molecular docking and in silico pharmacokinetic profiling, which provided information on their potential to act as antiviral agents and their suitability as therapeutics. The outcomes of docking showed that significant differences in the binding affinities of the two compounds to the viral targets were found. Cinnamic acid exhibited maximum affinity with NS5 (−5.970 kcal/mol) and capsid protein (−5.755 kcal/mol) which was mainly due to conventional hydrogen bonding and five influence of five interactions with the residues in the active or regulatory sites. These results are in line with earlier reports, including those of Zainal et al., which reported that cinnamic acid derivatives preferentially bind flavivirus polymerases via aromatic and polar interactions [11].

The RNA-dependent RNA polymerase of DENV is called NS5; it contains a number of conserved domains that take care of RNA elongation and 5-RNA capping [22]. Our simulations showed that cinnamic acid bound in the catalytic cleft, created hydrogen bonds with polar residues (Ser710), and formed electrostatic stabilization through the Arg729 and Asp540, which suggested that cinnamic acid could inhibit the enzyme in a competitive manner. Also, the aromatic ring of the cinnamic acid was involved in π-p stacking with Tyr607, which is an essential residue in the correct positioning of RNA templates [32].

Oleic acid, on the other hand, showed its highest binding affinity with the capsid protein (−6.150 kcal/mol), which is mainly caused by van der Waals forces and hydrophobic interactions with the aliphatic side chains of Leu66, Val77 and Ile80. The amphiphilic nature of the oleic acid enables it to insert in the hydrophobic pockets which are numerous in the dimer interface and the RNA-binding region of the capsid [33]. This binding molecule is stable with the results of Tsai et al. [34], who showed that fatty acids could interfere with capsid assembly and virion packaging by non-specific entrapment of hydrophobic molecules.

The cinnamic acid and oleic acid had weak interactions with the NS3 protease/helicase, suggesting poor geometric and electrostatic complementarity. In complex with NS2B, the NS3 protease domain will create a smaller and charged environment that is better fit to the basic or peptide-like inhibitors [23]. Cinnamic acid and oleic acid could not enter the active-site groove or interact with important catalyst residues (His51, Asp75, Ser135), and this is why their ability to inhibit this enzyme was low. The binding affinity (−4.275 kcal/mol) when docking cinnamic acid to the envelope protein was moderate. The ligand was stabilized by a hydrophobic contact and hydrogen bonds to the fusion loop and also occupied a shallow groove between the residues, Leu107, Thr198, and Asn153. Such interactions might also play a role in the conformational changes necessary in membrane fusion and are mentioned by Modis et al. [6], though their effect is less conspicuous than the interactions they have with NS5 or the capsid protein. The ADMET model showed that cinnamic acid has better drug-like properties than oleic acid. It forms a good molecular weight, moderate lipophilicity (LogP = 1.78) and good predicted oral bioavailability (0.91), as per the Rule of Five by Lipinski [35], which is why it has a chance of development. Moreover, cinnamic acid exhibited better solubility in aqueous solutions and greater intestinal permeability, as determined by the prediction of PAMPA and Caco-2, which were previously reported as well-permeable pharmacophores [36].

Conversely, oleic acid, which has a molecular weight of 282.47 Da and pronounced lipophilicity (LogP = 6.11), had low aqueous solubility and oral bioavailability. Even though it is not bound to lipids, thus making it an amphiphilic molecule, the compound has a very high plasma protein binding (>99) and the half-life is very long (26.8 h), which heightens apprehension towards bioaccumulation and systemic persistence [37]. Also, oleic acid is metabolized by several isoforms of cytochrome P450 (such as CYP1A2, CYP2C9, CYP3A4) and, therefore, the metabolic liability of oleic acid is higher and it can be likely to cause an increased risk of drug–drug interactions or metabolic toxicity [17].

Cinnamic acid was found to be less toxicological to oleic acid at various parameters. It had low hERG channel inhibition (0.01) and predicted mutagenicity which aligned with prior toxicogenomic evaluations [38]. However, its high predicted DILI score (0.91) suggests that it is experiencing hepatocellular stress, which needs additional hepatocyte-based study. In comparison, oleic acid had intermediate hERG inhibition (0.30) and a significantly elevated carcinogenicity index (0.61) in accordance with previous findings that long-chain unsaturated fatty acids cause oxidative stress-induced DNA damage in some circumstances [39]. Oleic acid was also found to cause more severe skin sensitization and mitochondrial potential disruption, which is indicative of wider off-target liabilities [40].

Receptor binding analysis showed that the two compounds both interact with nuclear receptors like PPAR 7 and NRF2. The activations of anti-oxidant and metabolic pathways were mildly activated by cinnamic acid, but were stronger in oleic acid, with PPAR7 being the most significant (0.33), as it may play a role in metabolic regulation but could also be involved in endocrine regulation [41,42].

Cinnamic acid and oleic acid have dual-targeting potential, which is an interesting pharmacological paradigm. The interaction with NS5 is an indication of a potential interference of genome replication, whereas the interaction with the capsid points to the interruption of RNA encapsidation. This compound, two-fold mechanism is similar to those of broad-spectrum antivirals, like suramin, that have both enzymatic and structural actions [43].

Oleic acid with less favorable pharmacokinetics binds well to the capsid by deep accommodation of hydrophobic pockets. The reason why this interaction is important is that disruption of the capsid can suspend virion maturation and genome package [44]. Capsid-targeted oleic acid with replication inhibitor agents such as cinnamic acid might prove to provide a synergistic treatment method. Moreover, cinnamic acid has good ADMET profile and drug-likeness, meaning that it has high lead optimization and derivatization potential. Recent research on cinnamate analogs has revealed that the potency has been enhanced through halogenation or amide replacements, which retain safety without compromising or affecting viral target binding [45]. Immune system and cytokines are a significant shareholder in a number of illnesses including diabetes [46] and hemorrhagic fever [47].

Although such in silico results are informative, there are some limitations. The lack of molecular dynamics simulations restricts evaluation of complicated stability in physiological circumstances. Inhibition potency cannot be proven by binding energies alone, and therefore biochemical measurements are necessary in order to prove enzymatic blockade. Also, the pre-predicted ADMET and toxicity profiles have to be validated in the cell models and in vivo. However, this paper offers a logical basis for developing further studies on cinnamic acid analogs, such as NS5/capsid inhibitor and oleic acid derivatives, as structural virucidal agents.

## 5. Limitation

The research is anchored in the use of computational modeling, which involves molecular docking, molecular dynamics simulation, and in silico prediction of ADMET. As much as these methods offer useful information on mechanistic basis, they do not directly validate biological activity or therapeutic activity. Docking scores and binding free energies are not conclusive results of enzymatic inhibition because they fail to capture any of the intricate intracellular circumstances, protein–protein interactions, or the dynamics of viral replication. The 50 ns simulations of the molecular dynamics were conducted at simplified system conditions, which might not necessarily reflect long-term conformational change, membrane-associated effects (especially of the envelope protein), or the entire viral replication complex. In addition, ADMET predictions are based on algorithm-driven models that are not always realistic of in vivo pharmacokinetics, metabolism, or toxicity pattern. The other limitation is there is no experimental validation. No animal models, viral replication studies, enzymatic inhibition assays, or cytotoxicity assays were done to confirm the desired antiviral activity. Also, GC–MS analysis indicates relative abundance in the extract but does not determine biological potency or their synergism as constituents. Notably, experimental analysis of the possible side effects of cinnamic acid and oleic acid was not done. Although in silico estimates indicated an acceptable safety profile of cinnamic acid and moderate liabilities of oleic acid (e.g., high lipophilicity and plasma protein binding), there are in vitro cytotoxicity assays, hepatotoxicity assays, cardiotoxicity screening (e.g., hERG channel testing), and long-term in vivo toxicity experiments required to confirm these results. The lipophilicity of oleic acid and its long half-life might be associated with concerns about bioaccumulation and its effects on the metabolic load, but cinnamic acid could be the subject of a reconsideration because of the possible hepatic stress indicators of predictive models. Thus, before clinical translation can be considered, extensive biochemical, cellular, pharmacokinetic and toxicological studies need to be conducted.

## 6. Conclusions

The present comparative in silico study elucidated the binding behavior and pharmacokinetic profiles of cinnamic acid and oleic acid, two major constituents of Cinnamomum verum, against four critical dengue virus (DENV) proteins—NS5, NS3, envelope, and capsid. Cinnamic acid exhibited superior docking affinity and specificity toward NS5 RNA-dependent RNA polymerase (−5.970 kcal/mol) and capsid protein (−5.755 kcal/mol), facilitated by hydrogen bonding and π–π stacking interactions, especially with polar and aromatic residues in the catalytic or RNA-binding domains. These interactions indicate a high potential to disrupt viral replication and assembly processes. Oleic acid demonstrated the best binding affinity to the capsid protein (−6.150 kcal/mol) through deep hydrophobic accommodation and stabilizing van der Waals interactions, in which its affinity to NS5 was moderate and largely due to lipophilic contacts. The ligands weakly interacted with NS3 protease/helicase, which means that their structures are chemically and structurally incompatible. Each compound was moderately bound to the envelope protein, and there was no adequate structural anchoring of its interaction to enable its strong antiviral effect. By pharmacokinetics, cinnamic acid showed a better aqueous solubility, oral bioavailability, and metabolic stability, and reduced carcinogenic risk, which is in favor of its drug-likeness and drug safety. Oleic acid, on the contrary, had greater metabolic liability, P-glycoprotein binding, and carcinogenicity and lower lipid affinity and capsid-targeting capacity. To conclude, despite the promising outlook of cinnamic acid as a multi-target antiviral against DENV, cinnamic acid would be a valuable candidate antiviral due to its ability to address NS5 and capsid proteins with optimal ADMET characteristics. Oleic acid has the potential of structural interference with the capsid but must not be used without caution because of the pharmacokinetic and toxicological disadvantages. These findings lay the groundwork for further experimental validation and structure-based optimization of cinnamic acid derivatives for anti-dengue therapy.

Future research ought to concentrate on experimental adherence to NS5 polymerase and capsid assembly interference based on enzymatic and cell-based antiviral design on assorted DENV serotypes. To improve the potency and selectivity of cinnamic acid derivatives, structure–activity relationship (SAR) optimization should be used to minimize or eliminate the possible risk of hepatotoxicity. In the case of oleic acid, pharmacokinetic suitability can be enhanced by structural modification in a way that reduces lipophilicity and plasma protein binding. Also, further computations of free energy and more extended molecular dynamics simulations are suggested to improve the prediction of bindings before performing in vitro and in vivo studies.

## Figures and Tables

**Table 1:** Selected dengue virus proteins with their biological functions and corresponding PDB identifiers.

No.	Protein Name	Biological Function	PDB ID
1	NS5 RNA-dependent RNA polymerase (RdRp)	Catalyzes replication of the viral RNA genome	5K5M
2	NS3 Protease/Helicase	Facilitates proteolytic cleavage and helicase activity during viral protein maturation	2FOM
3	Envelope protein (E protein)	Mediates viral attachment and membrane fusion with host cells	1OKE

**Table 2:** Chemical constituents of cinnamon extract based on GC-MS Analysis.

Peak	Active Compounds	Area (%)	RT (min)
1	2-Propenoic acid, 3-phenyl-, methyl ester	1.63	11.896
2	Cinnamic acid	85.92	13.520
3	Hexadecanoic acid, methyl ester	0.88	20.172
4	Pentadecanoic acid	1.05	20.733
5	9,12-Octadecadienoic acid, methyl ester	0.61	22.370
6	9-Octadecenoic acid, methyl ester	2.10	22.468
7	Oleic acid	5.33	23.029
8	Octadecanoic acid	0.68	23.283
9	Methanamine, N,N-difluoro	0.70	23.557
10	Tetracosane	1.09	24.666

**Table 3:** Computed calculated binding affinities (kcal/mol) of cinnamic acid with DENV target proteins with AutoDock Vina.

Model	A: (5k5m, NS5)	B: (2fom, NS3)	C: (1oke, Envelope)
1	−5.970	−3.369	−4.275
2	−5.680	−3.327	−3.643
3	−5.499	−3.319	−3.640
4	−5.168	−3.287	−3.600
5	−4.749	−3.112	−3.508
6	−4.735	−3.100	−3.493
7	−4.721	−2.852	−3.238
8	−4.672	−2.847	−3.190
9	−4.503	−2.838	−3.134
10	−4.427	−2.794	−3.036
11	−4.326	−2.697	−3.001
12	−4.268	−2.285	−2.846
13	−4.249	−2.283	−2.801
14	−3.813	−2.139	−2.749
15	−3.533	−2.102	−2.729
16	−3.468	−2.094	−2.666
17	−3.444	−2.010	−2.537
18	−3.404	−1.961	−2.309
19	−3.272	−1.943	−2.260
20	—	−1.940	−2.203

**Table 4:** Calculated binding affinities (kcal/mol) of oleic acid with various protein targets using AutoDock Vina.

Model	A: (5k5m, NS5)	B: (2fom, NS3)	C: (1oke, Envelope)
1	−5.209	−2.602	−3.188
2	−4.732	−2.598	−3.001
3	−4.701	−2.597	−2.958
4	−4.677	−2.553	−2.903
5	−4.659	−2.524	−2.888
6	−4.642	−2.460	−2.825
7	−4.620	−2.456	−2.760
8	−4.602	−2.430	−2.756
9	−4.553	−2.398	−2.627
10	−4.551	−2.306	−2.599
11	−4.507	−2.254	−2.530
12	−4.448	−2.185	−2.466
13	−4.426	−2.105	−2.403
14	−4.359	−2.071	−2.394
15	−4.286	−2.065	−2.348
16	−4.216	−1.964	−2.338
17	−4.183	−1.877	−2.241
18	−4.063	−1.872	−2.231
19	−3.958	−1.833	−2.220
20	−3.875	−1.639	−2.193

**Table 5:** Physicochemical properties.

Property	Cinnamic Acid	Oleic Acid
Molecular Weight (Da)	148.16	282.47
LogP	1.78	6.11
H-Bond Acceptors	1	1
H-Bond Donors	1	1
Lipinski Violations	0	1
QED	0.65	0.29
TPSA (Å^2^)	37.30	37.30

**Table 6:** Absorption and distribution parameters.

Property	Cinnamic Acid	Oleic Acid
Human Intestinal Absorption	0.99	1.00
Oral Bioavailability	0.91	0.66
Aqueous Solubility (log mol/L)	−2.36	−5.62
Cell Permeability	−4.58	−5.07
BBB Penetration	0.77	0.82
Plasma Protein Binding (%)	92.47	100
Volume of Distribution (L/kg)	4.61	5.33

**Table 7:** Metabolism (CYP interaction profile).

CYP Parameter	Cinnamic Acid	Oleic Acid
CYP1A2 Inhibition	Low	Moderate
CYP2C19 Inhibition	Low	Moderate
CYP2C9 Inhibition	Low	Moderate
CYP2D6 Inhibition	Very Low	Moderate
CYP3A4 Inhibition	Negligible	Low
CYP2C9 Substrate	Yes	Yes
CYP3A4 Substrate	No	Moderate

**Table 8:** Excretion parameters.

Property	Cinnamic Acid	Oleic Acid
Half-Life (h)	Short	26.8
Hepatocyte Clearance	Low	High
Microsomal Clearance	3.22	28.69

**Table 9:** Toxicological profile.

Endpoint	Cinnamic Acid	Oleic Acid
hERG Blocking	Very Low	Moderate
Mutagenicity	Low	Low
DILI Risk	High	Moderate
Carcinogenicity	Moderate	High
LD_50_	1.58	1.34
PPARγ Activation	Low	High

## Data Availability

The article and its Supplementary Materials have all the necessary information to support the main results of this research.

## Supplementary Materials

The following supporting information can be downloaded at https://www.mdpi.com/article/10.3390/idr18020026/s1, Supplementary S1: Comparative Structural Docking Analysis of Positive Control (Ribavirin) Against Dengue Virus Proteins; Supplementary S2: Molecular Dynamics Interaction Analysis of Cinnamic Acid with Target Proteins; Supplementary S3: Integrated Molecular Dynamics Analysis of the Oleic Acid with Target Proteins.
