# Supplementary material for: Comparative Molecular Docking and Pharmacokinetic Profiling of Cinnamic Acid and Oleic Acid from Cinnamomum verum as Potential Inhibitors of Dengue Virus Proteins
Source: Infect Dis Rep. 2026 Mar 26;18(2):26. doi: 10.3390/idr18020026 (PMC13116094; doi:10.3390/idr18020026)
Supplement: Supplementary file 1 [file idr-18-00026-s001.zip › Supplementary S1 Comparative Structural Docking Analysis of Positive Control (Ribavirin) Against Dengue Virus Proteins .pdf]

Result docking of positive control in supplementary 1.positive control Ribavirin (PubChem CID: 37542

Query

Ligand C1=NC(=NN1[C@H]2[C@@H]([C@@H]([C@H](O2)CO)O)O)C(=O)N

Target 5k5m_modified.pdb

Method AutoDock Vina

Date February 18, 2026, 2:40 pm UTC

Parameters:

Box center: -14 - -40 - -18 Sampling exhaustivity: 16

Box size: 20 - 20 - 20

If you publish these results, please, cite the following papers:

Bugnon M, Röhrig UF, Goullieux M, Perez MAS, Daina A, Michielin O, Zoete V. SwissDock 2024: major enhancements for small-molecule docking with Attracting Cavities and AutoDock Vina. *Nucleic Acids Res.* **2024**

Eberhardt J, Santos-Martins D, Tillack AF, Forli S.. AutoDock Vina 1.2.0: New Docking Methods, Expanded Force Field, and Python Bindings. *J. Chem. Inf. Model.,* **2021**

Model	Calculated affinity (kcal/mol)
1	-5.559
2	-5.536
3	-5.536
4	-5.525
5	-5.498
6	-5.450
7	-5.431
8	-5.410
9	-5.374
10	-5.337
11	-5.261
12	-5.235
13	-5.227
14	-5.197
15	-5.169
16	-5.150

Model	Calculated affinity (kcal/mol)
17	-5.127
18	-5.062
19	-5.036
20	-4.827

Ligand C1=NC(=NN1[C@H]2[C@@H]([C@@H]([C@H](O2)CO)O)O)C(=O)N

Target 2fom_modified.pdb

Method AutoDock Vina

Date February 18, 2026, 2:42 pm UTC

Parameters:

Box center: -1 - -13 - 16 Sampling exhaustivity: 16

Box size: 20 - 20 - 20

If you publish these results, please, cite the following papers:

Bugnon M, Röhrig UF, Goullieux M, Perez MAS, Daina A, Michielin O, Zoete V. SwissDock 2024: major enhancements for small-molecule docking with Attracting Cavities and AutoDock Vina. *Nucleic Acids Res.* **2024**

Eberhardt J, Santos-Martins D, Tillack AF, Forli S.. AutoDock Vina 1.2.0: New Docking Methods, Expanded Force Field, and Python Bindings. *J. Chem. Inf. Model.,* **2021**

Model	Calculated affinity (kcal/mol)
1	-4.662
2	-4.186
3	-4.135
4	-4.079
5	-4.062
6	-3.975
7	-3.957
8	-3.929
9	-3.914
10	-3.889
11	-3.830

Model	Calculated affinity (kcal/mol)
12	-3.764
13	-3.729
14	-3.664
15	-3.642
16	-3.624
17	-3.614
18	-3.519

Ligand C1=NC(=NN1[C@H]2[C@@H]([C@@H]([C@H](O2)CO)O)O)C(=O)N

Target 1oke_modified.pdb

Method AutoDock Vina

Date February 18, 2026, 2:45 pm UTC

Parameters:

Box center: -14 - 69 - 24 Sampling exhaustivity: 16

Box size: 20 - 20 - 20

If you publish these results, please, cite the following papers:

Bugnon M, Röhrig UF, Goullieux M, Perez MAS, Daina A, Michielin O, Zoete V. SwissDock 2024: major enhancements for small-molecule docking with Attracting Cavities and AutoDock Vina. *Nucleic Acids Res.* **2024**

Eberhardt J, Santos-Martins D, Tillack AF, Forli S.. AutoDock Vina 1.2.0: New Docking Methods, Expanded Force Field, and Python Bindings. *J. Chem. Inf. Model.,* **202**

Model	Calculated affinity (kcal/mol)
1	-4.243
2	-3.970
3	-3.924
4	-3.913
5	-3.868
6	-3.848
7	-3.688
8	-3.683
9	-3.642

Model	Calculated affinity (kcal/mol)
10	-3.607
11	-3.562
12	-3.558
13	-3.555
14	-3.525
15	-3.507
16	-3.505
17	-3.498
18	-3.424
19	-3.420
20	-3.394

Ligand NC(=O)C1=NN(C=N1)[C@@H]1O[C@H](CO)[C@@H](O)[C@H]1O

Target 1r6r_modified.pdb

Method AutoDock Vina

Date February 18, 2026, 2:45 pm UTC

Parameters:

Box center: 1 - -6 - -1 Sampling exhaustivity: 16

Box size: 20 - 20 - 20

If you publish these results, please, cite the following papers:

Bugnon M, Röhrig UF, Goullieux M, Perez MAS, Daina A, Michielin O, Zoete V. SwissDock 2024: major enhancements for small-molecule docking with Attracting Cavities and AutoDock Vina. *Nucleic Acids Res.* **2024**

Eberhardt J, Santos-Martins D, Tillack AF, Forli S.. AutoDock Vina 1.2.0: New Docking Methods, Expanded Force Field, and Python Bindings. *J. Chem. Inf. Model.*, **2021**

Model	Calculated affinity (kcal/mol)
1	-4.617
2	-4.591
3	-4.508
4	-4.469
5	-4.460

Model	Calculated affinity (kcal/mol)
6	-4.425
7	-4.375
8	-4.371
9	-4.322
10	-4.308
11	-4.245
12	-4.234
13	-4.149
14	-4.122
15	-4.032
16	-4.005
17	-3.994
18	-3.990
19	-3.949
20	-3.904
