# Supplementary material for: Comparative Molecular Docking and Pharmacokinetic Profiling of Cinnamic Acid and Oleic Acid from Cinnamomum verum as Potential Inhibitors of Dengue Virus Proteins
Source: Infect Dis Rep. 2026 Mar 26;18(2):26. doi: 10.3390/idr18020026 (PMC13116094; doi:10.3390/idr18020026)

Results

Interaction Counts

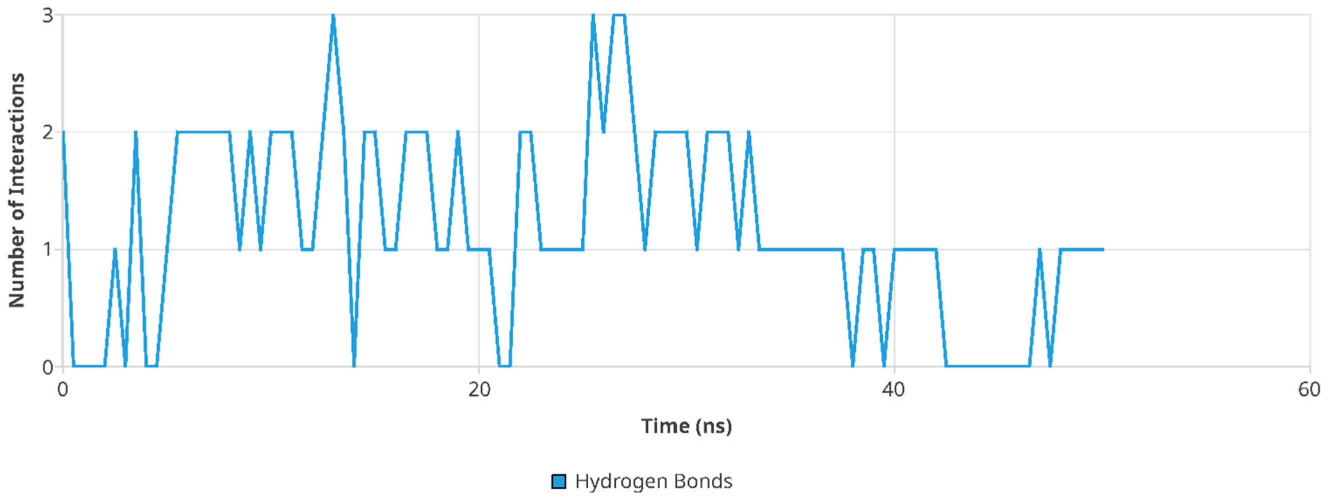

RMSD

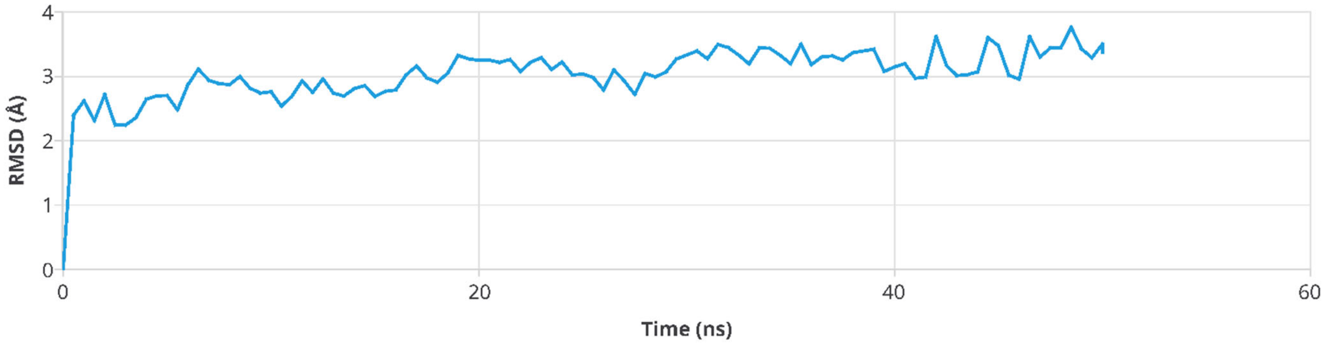

RMSF

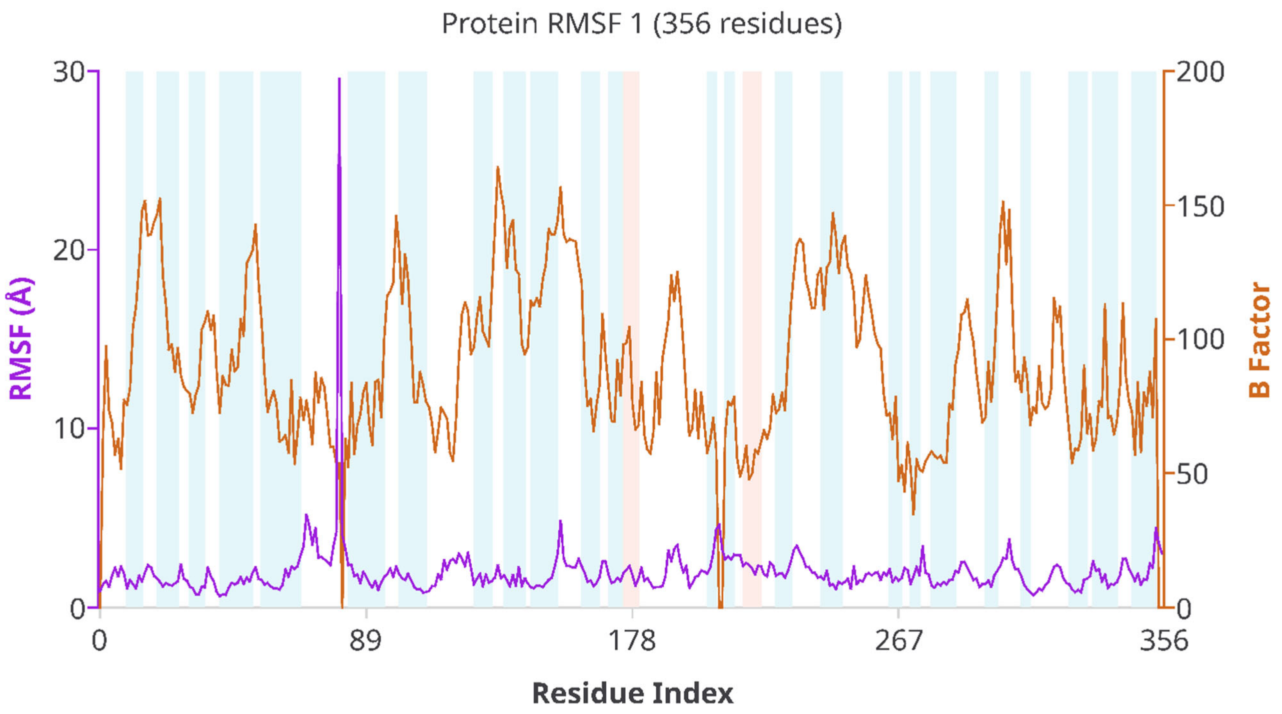

Radius of Gyration

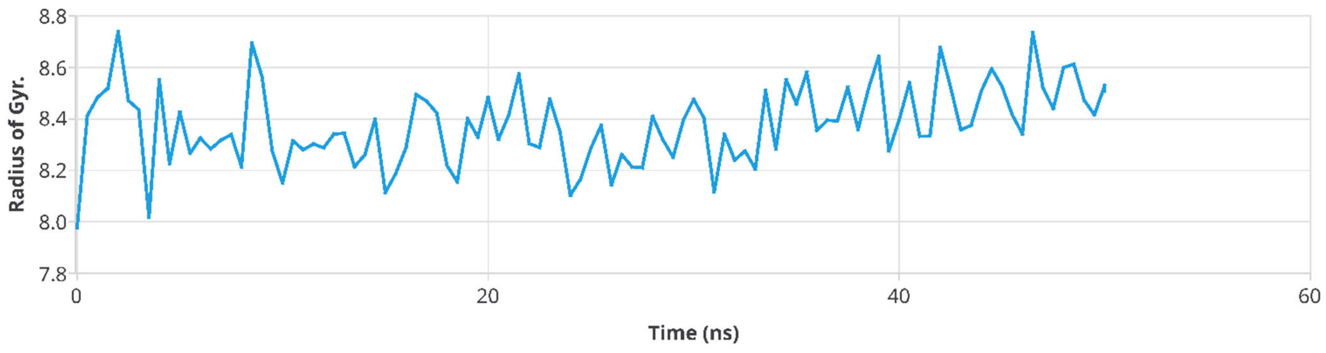

Molecular Surface Area

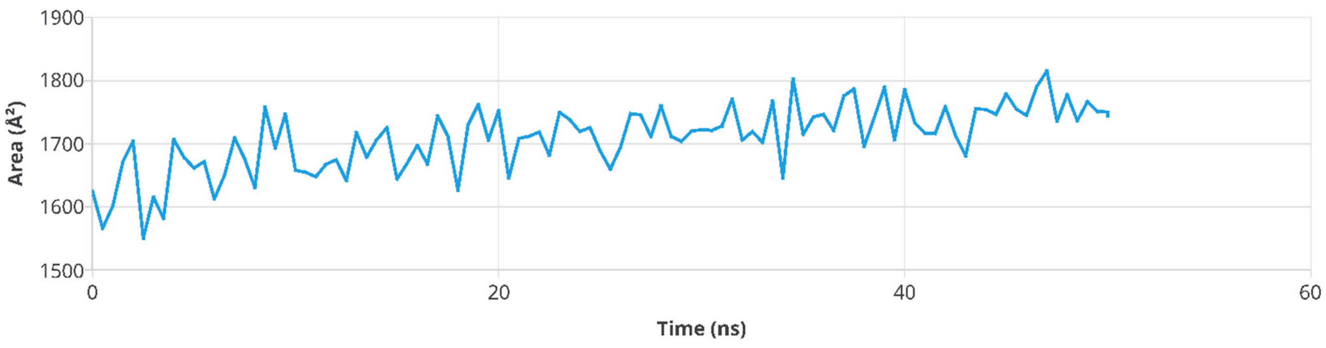

Polar Surface Area

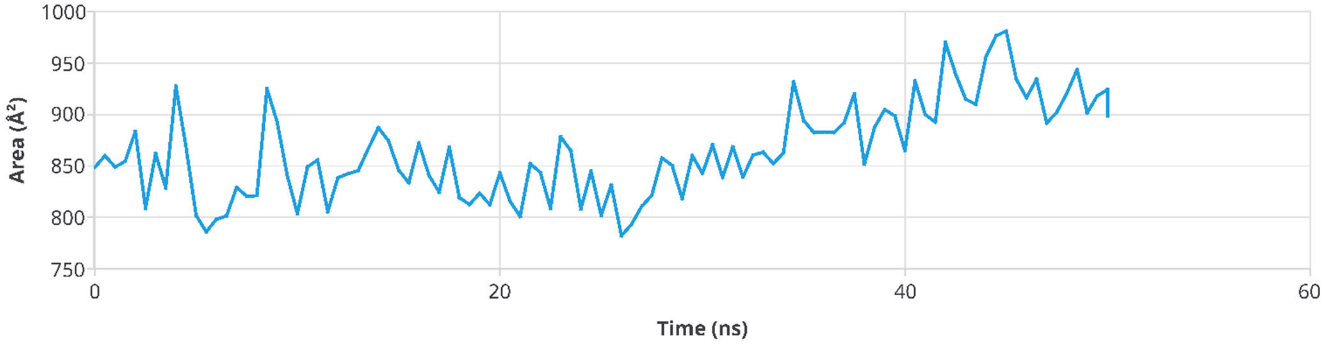

Radial Distribution Function (RDF)

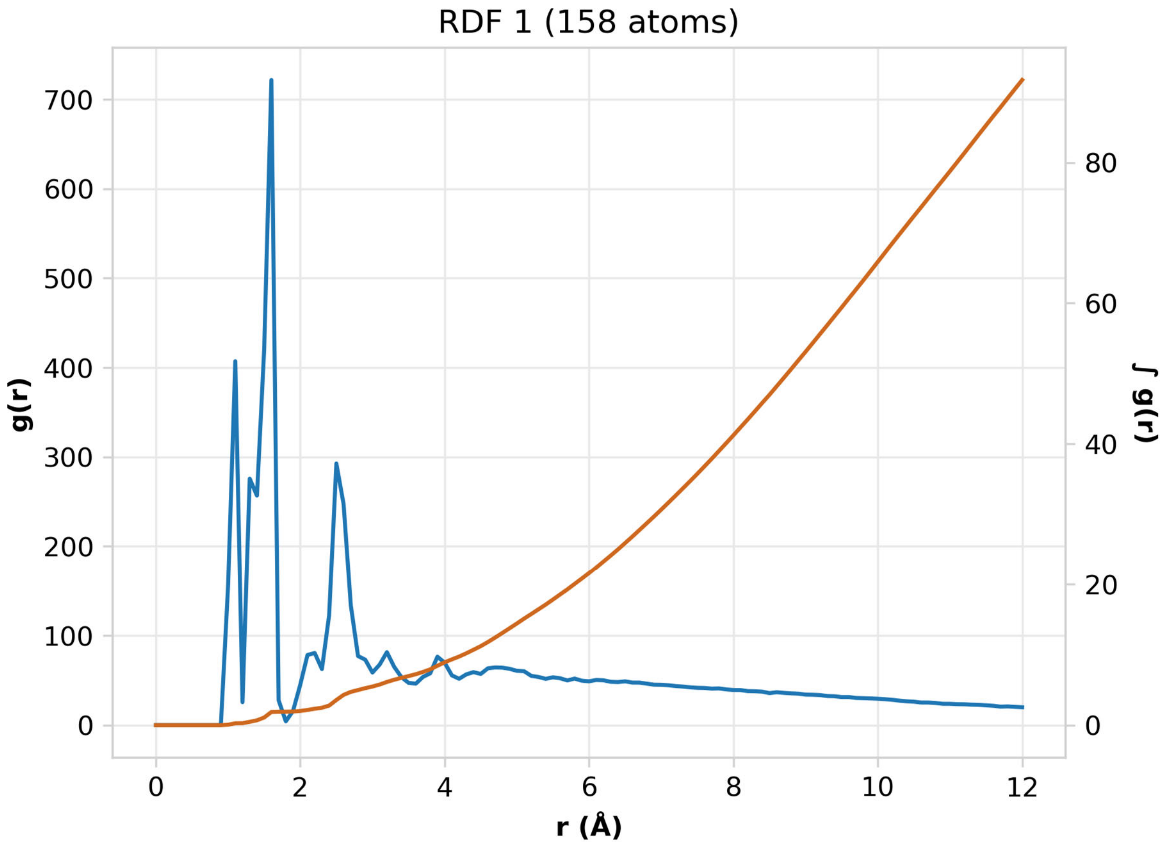

Solvent accessible surface area

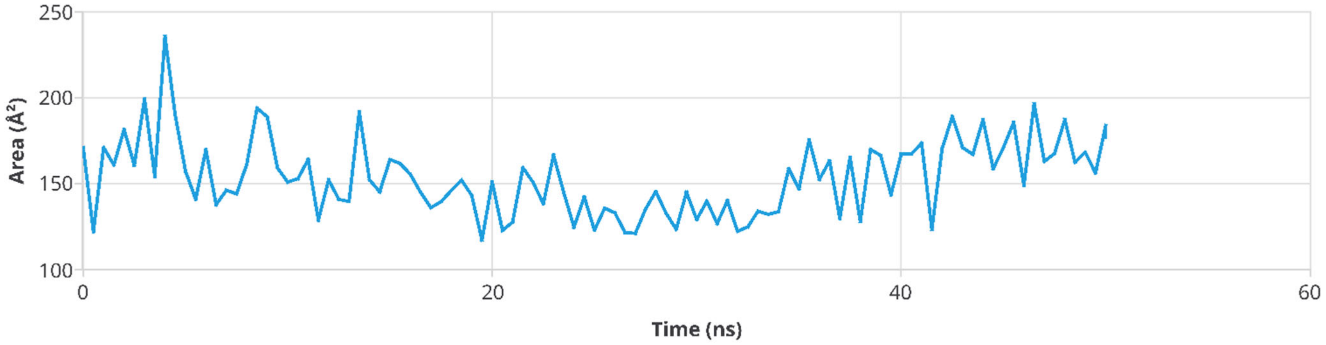

Supplement: Supplementary file 1 [file idr-18-00026-s001.zip › Supplementary S3 Integrated Molecular Dynamics Analysis of the Oleic Acid with Target Protiens/l2p3-1OKE.pdf]
